# Plant Diversity and Fertilizer Management Shape the Belowground Microbiome of Native Grass Bioenergy Feedstocks

**DOI:** 10.3389/fpls.2019.01018

**Published:** 2019-08-14

**Authors:** Daniel Revillini, Gail W. T. Wilson, R. Michael Miller, Ryan Lancione, Nancy Collins Johnson

**Affiliations:** ^1^Department of Biological Sciences, Northern Arizona University, Flagstaff, AZ, United States; ^2^Department of Biology, University of Miami, Coral Gables, FL, United States; ^3^Department of Natural Resource Ecology, Management, Oklahoma State University, Stillwater, OK, United States; ^4^Environmental Science Division, Argonne National Laboratory, Lemont, IL, United States; ^5^School of Earth, Sustainability, Northern Arizona University, Flagstaff, AZ, United States

**Keywords:** soil microbiome, switchgrass, rhizobacteria, arbuscular mycorrhizal fungi, resource availability, plant–microbial interaction

## Abstract

Plants may actively cultivate microorganisms in their roots and rhizosphere that enhance their nutrition. To develop cropping strategies that substitute mineral fertilizers for beneficial root symbioses, we must first understand how microbial communities associated with plant roots differ among plant taxa and how they respond to fertilization. Arbuscular mycorrhizal (AM) fungi and rhizobacteria are of particular interest because they enhance nutrient availability to plants and perform a suite of nutrient cycling functions. The purpose of this experiment is to examine the root and soil microbiome in a long-term switchgrass (*Panicum virgatum*) biofuel feedstock experiment and determine how AM fungi and rhizobacteria respond to plant diversity and soil fertility. We hypothesize that intra- and interspecific plant diversity, nitrogen fertilization (+N), and their interaction will influence the biomass and community composition of AM fungi and rhizobacteria. We further hypothesize that +N will reduce the abundance of nitrogenase-encoding nifH genes on the rhizoplane. Roots and soils were sampled from three switchgrass cultivars (Cave-in-Rock, Kanlow, Southlow) grown in monoculture, intraspecific mixture, and interspecific planting mixtures with either *Andropogon gerardii* or diverse native tallgrass prairie species. Molecular sequencing was performed on root and soil samples, fatty acid extractions were assessed to determine microbial biomass, and quantitative polymerase chain reaction (qPCR) was performed on nifH genes from the rhizoplane. Sequence data determined core AM fungal and bacterial microbiomes and indicator taxa for plant diversity and +N treatments. We found that plant diversity and +N influenced AM fungal biomass and community structure. Across all plant diversity treatments, +N reduced the biomass of AM fungi and nifH gene abundance by more than 40%. The AM fungal genus *Scutellospora* was an indicator for +N, with relative abundance significantly greater under +N and in monoculture treatments. Community composition of rhizobacteria was influenced by plant diversity but not by +N. *Verrucomicrobia* and *Proteobacteria* were the dominant bacterial phyla in both roots and soils. Our findings provide evidence that soil fertility and plant diversity structure the root and soil microbiome. Optimization of soil communities for switchgrass production must take into account differences among cultivars and their unique responses to shifts in soil fertility.

## Introduction

National initiatives to increase energy independence spurred interest in biofuel cropping systems and native feedstock options (Perlack et al., 2011). The use of tallgrass prairie plants, including switchgrass (*Panicum virgatum* L.) and big bluestem (*Andropogon gerardii* Vitman) as biofuel feedstocks can be both economical and ecologically beneficial (Parrish and Fike, 2005; Tilman et al., 2006; Yang et al., 2018). Native, perennial warm-season grasses are broadly adapted to North America, are well-suited to grow on marginal soils, and can maintain high levels of productivity with minimal fertilization. They also provide additional ecosystem services including erosion control, increased C sequestration (Adkins et al., 2016; Hungate et al., 2017), reduction of nutrient leaching, and wildlife habitat (Parrish and Fike, 2005).

Studies have determined the importance of plant diversity on productivity in native feedstock cropping systems (Tilman et al., 2006; Mangan et al., 2011; Morris et al., 2016), but considerably less is understood about the interactions between plants and their microbial partners in roots and soil. Switchgrass and big bluestem are highly responsive to arbuscular mycorrhizal (AM) fungi (Chagnon and Bradley, 2013; Johnson et al., 2010) and rhizobacteria, though substantially less is known about their interactions with rhizobacteria (Brejda et al., 1998; Mao et al., 2014). Microbial symbionts likely provide the nutritional support needed for these native grasses to thrive in low-fertility soils and under drought. These beneficial plant–microbial relationships are locally adapted (Johnson et al., 2010; Revillini et al., 2016) and can be actively promoted in a plant taxa-specific manner (Berg et al., 2014; Agler et al., 2016). An understanding of the belowground microbiome on roots and in soils is necessary to begin to understand how microbial symbioses can facilitate and maintain high plant yields in any cropping system (Johansson et al., 2004; Schlaeppi and Bulgarelli, 2015; Alori et al., 2017). This will be particularly useful for native biofuel feedstocks, as they are promoted for cultivation on marginal lands no longer suitable for row cropping and where soil microbes will have likely been altered by conventional agricultural practices (Hart and Trevors, 2005; Verbruggen et al., 2015). Furthermore, given the relatively recent breeding history of native grasses for biofuel production, it is important to begin to understand the roles of plant cultivar, plant diversity, and fertilization regime on the microbial communities that nutritionally support their productivity on marginal soils (Busby et al., 2017).

Much like the human microbiome (Gilbert et al., 2018) and the plant surface microbiome (Vacher et al., 2016), root and soil-borne microbial communities play significant roles in host productivity and nutrient cycling from a variety of mechanisms (Bakker et al., 2018), but they also influence host-plant community dynamics (Bardgett and van der Putten, 2014; Vandenkoornhuyse et al., 2015; van der Heijden et al., 2016). The functioning of AM fungi and rhizobacteria can be influenced by both the relative availability of resources [e.g. soil nitrogen (N) and phosphorus (P)], functional traits within plant communities, and the feedbacks between plant and microbial communities (Larimer et al., 2010). Feedbacks between plants and soil microbial communities over seasons and generations are common (van der Putten et al., 2013) and can influence plant diversity, disease resistance, and resource-use efficiency (Bever, 2015; Mariotte et al., 2017). Plants can preferentially allocate photosynthate to microbial symbionts that provide the most resources (Bever et al., 2009; Kiers et al., 2003; Ji and Bever, 2016), and in turn, AM fungi and rhizobial bacteria may reciprocate by provisioning the most P or N to the most generous host plants (Kiers et al., 2011). Theory predicts that these resource allocation relationships, which are recognized most strongly in nutrient-limited systems (Semchenko et al., 2018), can influence the composition and functionality of microbial symbiont communities found in soil through preferential selection of the most beneficial partners (Kiers and Denison, 2008; Bever, 2015). The realized abundance and composition of microbial communities under different environmental contexts and management regimes are influenced by the interplay of preferential selection for more beneficial nutritional partners versus the accumulation of commensalistic or parasitic organisms (Adesemoye et al., 2009; Wei et al., 2013; Rubin et al., 2017).

Switchgrass cultivars may differ in the degree to which they form symbiotic associations to acquire essential resources (Brejda et al., 1998; Bouton, 2007; Bauer et al., 2012). Consequently, the diversity of feedstock planting mixtures, including intraspecific diversity among switchgrass cultivars, may be expected to influence the abundance and community composition of bacteria and AM fungi. This would be a likely consequence of the extended plant phenotype, which incorporates plant-associated microbiota critical to plant productivity and survival into the observed “plant” phenotype. This concept integrates the direct and often reciprocal effects among plant and microbial communities that can be structured by resource transfer mechanisms including optimal resource allocation (Berg et al., 2014; Vandenkoornhuyse et al., 2015). In a recent study, switchgrass genotypes and ecotypes were found to strongly drive differences in bacterial and fungal community composition, documenting the ability of switchgrass to maintain host-selected microbiomes (Singer et al., 2019). Optimal resource allocation between plants and microbial symbionts occurs when the investment of resources is targeted to specific structures, taxa, or functions that most strongly alleviate the limitations to productivity. This has been experimentally shown in AM fungal and legume–rhizobia mutualisms (Johnson, 2010; Kiers et al., 2011; Zheng et al., 2014). Optimal allocation to microbial structures or taxa may be directed by functional requirements specific to switchgrass cultivars that exhibit a range of phenotypes. Switchgrass cultivars have different root architectures and respond differently to fertilization (Oates et al., 2016; Sprunger et al., 2017). Morris et al. (2016) showed that the most diverse planting mixtures provided consistently high yields of high-quality feedstock. Consequently, we hypothesized that microbial community composition and biomass may be influenced by the interplay of intra- and interspecific plant diversity and soil fertility.

Nitrogen fertilization (+N) is a standard but costly management practice for switchgrass plantings (Yang et al., 2018), and there is interest in developing management strategies to harness naturally occurring bacteria and fungi that promote plant health so that fertilizer inputs may be minimized (Bender et al., 2016; Busby et al., 2017; Toju et al., 2018). Mutualistic legume–rhizobia interactions play an important role in N acquisition and cycling, but the vast majority of archaea and bacteria that promote plant growth in soils are “free-living” and not under the strict controls found in symbiotic relationships (Lugtenberg and Kamilova, 2009; Reed et al., 2011; Moore et al., 2015). Although it is well documented that AM fungi often contribute to plant uptake of N from soils (Govindarajulu et al., 2005; Hodge and Storer, 2015), they may also compete for N (Püschel et al., 2016) and not ameliorate N limitation of their host plants (Reynolds et al., 2005). The availabilities of P and N interact to determine AM fungal responses to +N (Johnson et al., 2003b; Johnson et al., 2015). When P is not in limited supply, +N likely decreases the abundance of both AM fungi and N-fixing bacteria as plants allocate more photosynthate to aboveground production rather than to nutritional mutualisms. In arable soils that often have sufficient levels of P, the biomass of AM fungi and the abundance of nifH genes (a proxy for N_2_-fixation) may be expected to decrease with +N as plants rely less on microbial N-cycling functions (Wei et al., 2013; Jach-Smith and Jackson, 2018). The fungal/bacterial (F/B) biomass ratio, a broad functional measure of microbial composition, may respond strongly to changes in nutrient availability and also change as a result of shifting plant allocation patterns (de Vries et al., 2006; Hannula et al., 2017). In many grassland studies, F/B ratio decreases with fertilization as fungal biomass declines significantly (Strickland and Rousk, 2010), and this response may be useful to understand the mechanisms of microbially mediated plant responses to N fertilization.

Baseline studies of communities of fungi and bacteria may reveal potentially important responses to plant diversity, N fertilization, and their interactions. For example, bacteria in the phylum *Verrucomicrobia* are very common in grassland soil (Bergmann et al., 2012; Fierer et al., 2013) and have been reported to be negatively correlated with soil N availability (Ramirez et al., 2012), but little is known about their functional role in soil ecology. Discovery of microbial indicator species or changes in relative abundance of particular taxa could indicate plant optimization of microbial communities with beneficial traits (Hart and Trevors, 2005; Azcón-Aguilar and Barea, 2015). Switchgrass cultivars can differ in the degree to which they respond to +N and to microbial mutualisms (Mao et al., 2014; Morris et al., 2016), so the diversity of feedstock planting mixtures and their responses to +N are likely to provide useful insights. The purpose of our study is to assess the effects of intra- and interspecific plant diversity and +N on the biomass and composition of communities of bacteria and AM fungi associated with both switchgrass roots and rhizosphere soils. We specifically tested the hypotheses that:

1) Switchgrass cultivars vary in the degree to which they support AM fungi and N-fixing bacteria and archaea.
2) Species richness of AM fungi should be greater with higher plant diversity (interspecific diversity > intraspecific diversity > monocultures).
3) Bacterial N-fixing activity should vary with intraspecific switchgrass diversity.

Additionally, we tested the hypotheses that N fertilization should:

4) Result in greater biomass allocation to aboveground production.
5) Reduce the biomass of AM fungi, N-fixing activity of bacteria, and decrease the F/B ratio.
6) Influence the community composition of AM fungi and bacteria, particularly in ways specific to switchgrass cultivar.

A robust evaluation of microbiome composition and the key members in plant–microbial interactions will provide a starting point for tests of specific functional relationships between soil microbial taxa and target crops (Bender et al., 2016). In this study, we analyzed microbial community composition to identify both indicator taxa and core bacterial and fungal communities that associate with different levels of plant diversity and fertilization to establish a baseline understanding of the extended plant phenotypes of switchgrass that are commonly used in biofuel feedstock experiments and production trials (Wright and Turhollow, 2010).

## Materials and Methods

### Study Site and Experimental Design

Experimental plots 36 m x 20 m were established in June 2008 at the Fermilab National Environmental Research Park in Batavia, IL, to compare the performance of switchgrass cultivars grown in monocultures, intraspecific mixture, and interspecific mixtures with big bluestem grass or with 10 other native prairie plants. The experiment includes three randomized complete blocks with split-plot treated with or without N fertilizer (+67 kg N ha^-1^ year^-1^ of 46-0-0 granular urea). Three switchgrass cultivars were planted in monoculture: Cave-in-Rock, a commercial upland cultivar; Kanlow, a commercial lowland cultivar; and Southlow, a regional upland cultivar in early breeding development. Southlow had slow initial establishment in monoculture at this experiment (Morris et al., 2016). In the switchgrass mixture treatment, the three cultivars were planted in equal proportions (33%) and were estimated to each be well-represented in switchgrass mixture. In the big bluestem mixture treatment, three switchgrass cultivars were planted in equal proportion with three cultivars of big bluestem. Finally, the prairie mixture treatment included equal proportions of the switchgrass and big bluestem cultivars (7% each) and ten additional common, native prairie grasses and forbs (**Supplemental Table 1**, and see Morris et al. (2016) for additional design details). Root samples were collected from three monocultures with two fertilization treatments, replicated six times for a total of 36 root samples. Soil samples were collected from all six planting treatments with two fertilization treatments, replicated six times for a total of 72 soil samples.

### Plant Biomass and Soil Nutrients

After the first killing frost (∼mid-November), aboveground biomass was harvested ∼15 cm above ground level, baled, and weighed using a hanging scale. Mean aboveground biomass from production years 2013–2015 was used to account for inter-annual variability. Mean percent change from unfertilized plots was calculated for fertilization responses as (Fertilized_mean_ – Unfertilized_mean_/Unfertilized_mean_)*100 and presented in figures, though statistical analyses were performed on raw data (**Table 1**). Root biomass was sampled from monoculture plots using soil root cores (5 cm diameter) to a depth of 15 cm. Three cores per plot were collected, the 0–5 and 5–15 cm portions from cores were separated, and respective portions were homogenized. Roots were separated from soil by rinsing with water over a 500 µm sieve, dried, and weighed. Root biomass was converted to g m^-2^ using soil bulk density, and root mass fraction was calculated as root biomass/root + shoot biomass (Wilsey and Wayne Polley, 2006). In 2013, three soil cores (2 cm × 15 cm) were collected from each plot, roots were removed, soils were homogenized, and extractable P was analyzed using the Mehlich-3 method (Mehlich, 1984). Inorganic N was analyzed following extraction using 1M KCl extraction and isotope ratio mass spectrometry to measure NO_3_^-^ and NH_4_^+^ (Burger and Jackson, 2003), which were combined for total N measures. Inorganic N was not measured in the soils collected from diverse prairie plots; therefore, statistical analyses do not include these data.

**Table 1:** Mean aboveground biomass (g m^-2^), root biomass (g m^-2^), available soil phosphorus (Mehlich P), and total available soil nitrogen (N; NO_3_^-^ + NH_4_^+^) from all applicable planting treatments under unfertilized or N-fertilized plots (+67 kg N ha^-1^ year^-1^).

Planting treatment	Fertilization	Aboveground biomass ( ± SD)	Root biomass ( ± SD)	Soil P ( ± SD); ppm	Soil N ( ± SD); ppm
*Monocultures*					
Cave-in-Rock	Unfertilized	621 (37)^a^	336.6 (77)^d^	6.65 (3.18)^ab^	9.05 (1.33)^b^
	Fertilized	**837 (9.4)^c^**	283.8 (53)^cd^	5.67 (0.33)^a^	**14.52 (2.86)^cd^**
Kanlow	Unfertilized	338 (8.7)^e^	249.4 (44)^bc^	5.28 (1.67)^a^	10.54 (0.84)^ab^
	Fertilized	**629 (16)^ab^**	**148.2 (12)^a^**	5.30 (1.24)^a^	12.79 (1.81)^ac^
Southlow	Unfertilized	588 (20)^a^	218.6 (30)^abc^	9.65 (3.34)^b^	9.42 (1.53)^ab^
	Fertilized	**697 (8.1)^b^**	193.9 (72)^ab^	6.15 (2.52)^ab^	**16.49 (3.61)^d^**
*Mixtures*					
Switchgrass	Unfertilized	613 (56)^a^	242.3 (16)^abcd^	5.36 (1.57)^ab^	9.09 (0.91)^ab^
	Fertilized	**836 (22)^c^**	184.1 (48)^abc^	5.40 (1.54)^ab^	**13.5 (1.35)^acd^**
Big bluestem	Unfertilized	795 (5.8)^c^	–	–	–
	Fertilized	**929 (27)^d^**	–	–	–
Prairie	Unfertilized	649 (79)^ab^	–	–	–
	Fertilized	**815 (43)^c^**	–	–	–

Mean values in bold indicate significant (p < 0.05) differences between fertilization treatments determined by ANOVA, and letters indicate differences from Tukey’s HSD post hoc of N-fertilization by planting treatment. Switchgrass = intraspecific mixture of the three switchgrass genotypes; big bluestem = interspecifc mixture of three switchgrass genotypes + three big bluestem genotypes; prairie = big bluestem mixture + 10 native grasses and forbs.

### Microbial Biomass

Biomass of AM fungi and bacteria was measured using signature fatty acids (Olsson et al., 1995). Phospholipid fatty acids (PLFAs) are constituents of biological membranes that can be used to estimate active biomass of bacteria and fungi, as biovolume and cell surface area are well correlated (Frostegård et al., 2011). Neutral lipid fatty acids (NLFAs) are basic storage products of many fungi and serve as the primary energy reserve in AM fungi (Sharma and Buyer, 2015). PLFAs and NLFAs were extracted from soils (n = 72) using an extraction outlined in Zogg et al. (1997). Soils cores were homogenized, freeze-dried, and then sent to Oklahoma State University (OSU) for lipid extractions. Briefly, total lipid extracts were separated into PLFAs and NLFAs using silicic acid chromatography, the fatty acids were cleaved from the glycerol backbone using KOH saponification, and the harvested fatty acids were methylated to form fatty acid methyl esters (FAMEs). The FAMEs were then analyzed by gas chromatography and mass selection detection using a gas chromatography mass spectrometry (GCMS) unit Agilent MS 5975C/GC 7890A. We utilized c:19 as an internal standard. For extraradical AM fungal biomass, we utilized the 16:1ω5c FAME biomarker for both PLFA and NFLA determination (Sakamoto et al., 2004). Biomarkers 18:2ω6,9c and 18:1ω9c were used to determine saprotrophic fungal biomass (Gray et al., 2011). Bacterial biomarkers were used to determine both Gram-negative and Gram-positive bacterial biomass following Frostegård and Bååth (1996). F/B ratio was calculated using the sums of all measured bacterial PLFA and fungal NLFA biomarkers.

### Sampling and Molecular Analysis of Roots and Soils

Root and soil samples were collected from the experimental plots in June 2015. Root samples were collected by “tracing” primary and lateral roots (<3mm diameter) below six switchgrass plants from each monoculture plot (n = 3). Six soil cores (2 cm x 15 cm) were taken randomly from within each sub-plot (16 m x 8 m) of all planting treatments. Root and soil samples were transported from the field to the laboratory in a cooler and frozen within four hours of collection. Samples were stored at -20°C until further processing for molecular analyses.

Next-generation sequencing was performed on DNA extracted from roots (n = 36) collected from monocultures of Cave-in-Rock, Kanlow, and Southlow switchgrass cultivars. The roots were not surface-sterilized, in order to retain an intact rhizoplane community along with endophytic bacteria. DNA was also extracted from homogenized soil cores (n = 72) from every plot for sequencing. DNA was extracted from roots and soils using the PureLink Microbiome DNA Purification Kit (Invitrogen, Carlsbad, CA, USA) following protocol from the “soil samples user guide.” Genomic DNA was observed by NanoDrop and then purified using magnetic beads in 18% Polyethylene glycol (PEG) to remove potential polymerase chain reaction (PCR) inhibitors. PCR was carried out utilizing the 515F-806R primers to amplify the V4 region of the 16S rRNA (Gilbert et al., 2014) to characterize bacterial/archaeal communities, and WANDA-AML2 primers to amplify the small subunit (SSU) region to characterize AM fungal communities (Lee et al., 2008; Dumbrell et al., 2011). This region was targeted for AM fungal communities as the taxonomy of this region is well-supported by the MaarjAM database (Öpik et al., 2010). DNA quantitation was performed using standard dsDNA quantitation for PicoGreen (Thermo Fisher Scientific, Inc., Waltham, MA, USA), and all samples were normalized to 2ng DNA/µL prior to pooling into 16S or SSU libraries. The libraries were purified, concentrated, and quantified using qPCR against Illumina DNA standards (Kapa Biosystems, Wilmington, MA). Samples were paired-end sequenced (2 x 250 mode for 16S and 2 x 300 mode for SSU) using the Illumina MiSeq desktop platform (Illumina, Inc., San Diego, CA, USA) in the Environmental Genetics and Genomics Laboratory at Northern Arizona University (NAU) (https://in.nau.edu/enggen/).

### NifH Gene Abundance

Abundance of the nifH gene was used as a proxy measure for N-fixation potential (Levy-Booth et al., 2014). Absolute nifH gene abundance was measured using qPCR with the targeted nifH amplicon primers IGK3/DVV. DNA from root samples (n = 36) free of PCR inhibitors (see above) was used for nifH qPCR, which was performed following a slightly modified method from Gaby and Buckley (2012). Briefly, reaction volumes were 20 µl, and 0.35 µM of each primer was used in each reaction. 40 cycles at annealing temperature of 56°C was used for initial PCR with IGK3/DVV. qPCR standards were created by first normalizing initial nifH PCR to 0.5 ng µl^-1^, nifH tailing with a 15x cycle PCR, and 18% PEG bead cleaning, and quantified using standard dsDNA quantitation with PicoGreen (Thermo Fisher Scientific, Inc., Waltham, MA, USA) prior to qPCR with QuantStudio 5 (Applied Biosystems, Waltham, MA, USA).

### Data Processing

All data processing methods were performed on both SSU and 16S data, unless specifically noted. Contaminating PhiX sequence was removed using the akutils *phix_filtering* command in akutils v1.2 (Krohn, 2016; https://github.com/alk224/akutils-v1.2). For bacterial data, read pairs were merged in akutils using the *join_paired_reads* command. Merged bacterial/archaeal data had an average length of 253 nt. Reads were not joined for AM fungal data and had an average length of 501 nt. Demultiplexing and quality filtering were carried out with the split_libraries_fastq.py command in QIIME 1.9.1 (Caporaso et al., 2010b) using a minimum quality threshold of q20, 0 bad characters allowed, and retaining only reads that satisfied these requirements for at least 95% of their length (-q 19 -r 0 -p 0.95). Chimeras were removed using the –uchime_ref option in vsearch 1.1.1 (Rognes et al., 2016) and the Gold reference database (http://drive5.com/uchime/gold.fa). One root sample was removed from downstream analyses due to low sequence count. Sequences were de-replicated on the first 100 bases using the prefix/suffix Operational taxanomic unit (OTU) picker in QIIME. OTU picking was performed *de novo* with Swarm (Mahé et al., 2014) at d4 resolution (∼98.4% similarity). Taxonomic identities were assigned with BLAST using default settings in QIIME 1.9.1 against the 97% Greengenes database (McDonald et al., 2012) for bacterial data and at 90% similarity against the MaarjAM database (Öpik et al., 2010) for AM fungal data. AM fungal taxa in the MaarjAM database are classified as virtual taxa (VT) but are referred to as OTUs in methods for brevity. outsequences were aligned using PyNAST (Caporaso et al., 2010a), and the resulting alignment was used to construct a phylogenetic tree with FastTree (Price et al., 2009). OTUs constituting less than 0.005% of the total data set were removed (Bokulich et al., 2012). OTU tables were rarefied to the lowest sample depth (3,449 for 16S; 3,484 for SSU) for α-diversity analyses. Relative abundance of taxa by treatments was analyzed for planting and fertilization treatments using the *group_significance.py* command in QIIME. Tests of ß-diversity and differential abundance were performed on OTU tables transformed by cumulative sum scaling (CSS) normalization (Paulson et al., 2013). Diversity analyses were conducted with the *core_diversity* command in akutils unless specified.

### Statistical Analyses

Analyses were only performed on plots with paired sequencing and physical plant or soil measurements, leaving n = 35 for root samples and n = 72 for soil samples. Blocking did not have effects on any measures across all statistical analyses in this study and therefore was not presented. Plant biomass, AM fungal biomass (PLFA/NLFA), and nifH gene abundance were tested using nonparametric Kruskal–Wallis, or parametric analysis of variance (ANOVA) and Tukey’s honestly significant difference (HSD) *post hoc* (*aov* and *TukeyHSD* in R, respectively) to determine +N, plant treatment, and +N * plant treatment interactions. All data were log-transformed when necessary to meet normality assumptions. Bacterial and fungal community α-diversities were compared by nonparametric Student’s T-test using rarefied OTU tables. Log_2_ fold change from control after +N of rarefied fungal and bacterial taxonomic relative abundance was calculated using DESeq2 (Love et al., 2014). Briefly, log_2_ fold change data presented are taxa with relative abundance that was significantly different after +N using the likelihood ratio test (LRT) where p < 0.05. Indicator species analyses were performed with rarefied OTU tables for each treatment, or treatment interaction using both the indicator value (IndVal = fidelity and relative abundance) and point-biserial correlation (r_pb_) coefficient with 9,999 permutations with multipatt function from the *indicspecies* package in R (De Cáceres and Legendre, 2009), and results are only presented when Benjamini–Hochberg false discovery rate (fdr)–corrected p < 0.05 (De Cáceres et al., 2010). The compute_core_microbiome.py command to was used to identify the core microbiome communities, defined here as OTUs that are present in 50% of samples for a treatment or treatment combination. Differences in bacterial and fungal ß-diversity were assessed by Permutational ANOVA (PERMANOVA) (Anderson, 2001) using weighted and unweighted UniFrac (Lozupone and Knight, 2005) for bacterial data and Bray–Curtis dissimilarity for AM fungal data. PERMANOVA was initially performed across all samples (n = 107) to determine differences in root and soil microbial communities with the respective diversity metrics. A pairwise PERMANOVA function (https://github.com/pmartinezarbizu/pairwiseAdonis) was used to determine microbial community dissimilarities within planting treatments under unfertilized or +N plots. Statistical significance from pairwise PERMANOVA was indicated when Bonferroni-corrected p < 0.05. All analyses were performed in R and with the use of the NAU High Performance Computing cluster (https://nau.edu/high-performance-computing/overview/).

## Results

### Plant Biomass and Soil Nutrients

**Figure 1:**
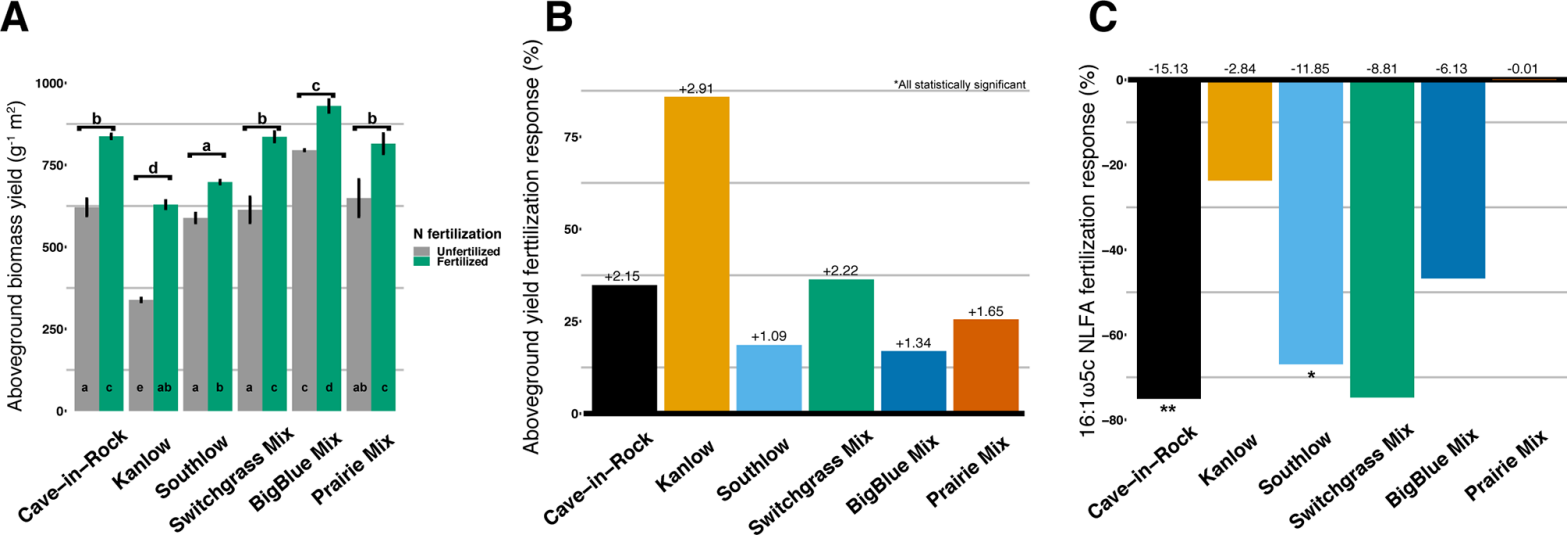
Total aboveground plant biomass (g m^2^) from each planting mixture under unfertilized or N-fertilized conditions **(A)** and aboveground biomass response to N fertilization (percent change) for all planting mixtures **(B)**. Arbuscular mycorrhizal (AM) fungal neutral lipid fatty acid (NLFA) response (percent change) to N fertilization for all planting mixtures **(C)**. Zero bar **(B**, **C)** represents unfertilized means per planting treatment. Values above bars in B are mean increases in plant biomass (Mg ha^-1^), and those in C are mean decreases in AM fungal biomass (nmol g^-1^ soil) per planting mixture. Letters indicate significant differences from Tukey’s HSD *post hoc* testing. Asterisks indicate significant differences from ANOVA (**p < 0.01, *p < 0.05).

Aboveground biomass from all planting mixtures was significantly greater in +N plots compared to unfertilized plots, with increases in monocultures of 35% for Cave-in-Rock, 85% for Kanlow, 18.5% for Southlow, 16.9% in big bluestem, and 25.5% in prairie mixtures (**Table 1**, **Figures 1A–B**). The big bluestem planting mixture produced higher biomass yields than all other planting treatments in both the unfertilized and +N plots (**Table 1**, **Figure 1A**). Root biomass was greater in unfertilized plots than fertilized plots (F_1,12_ = 5.62, p < 0.05) and different between switchgrass monocultures (F_2,12_ = 8.1, p = 0.005; **Supplemental Figure 1**). Root biomass was greater in Cave-in-Rock than Kanlow or Southlow (p < 0.01 and p < 0.05, respectively; **Supplemental Figure 1A**) and decreased with +N in Kanlow (p = 0.039). Root mass fraction was lower in +N plots (F_1,32_ = 19.2, p < 0.005; **Supplemental Figure 1B**) and higher under Kanlow than Southlow (F_3,18_ = 5.5, p = 0.02; **Supplemental Figure 1B**). In Kanlow, root mass fraction decreased in +N plots (p < 0.01; **Supplemental Figure 1B**). Soil available P was lower under +N (F_1,34_ = 4.1, p = 0.0508; **Table 1**) and higher under Southlow than Kanlow (F_3,34_ = 3.8, p = 0.018; **Table 1**). There was no fertilization–by–planting treatment interaction for soil P. Soil N (NH_4_^+^ + NO_3_^-^; ppm) increased with +N overall (F_1,34_ = 67.6, p < 0.001; **Table 1**) and within Cave-in-Rock and Southlow monocultures and switchgrass mixture plots (**Table 1**).

### AM Fungal and Bacterial Biomass

**Figure 2:**
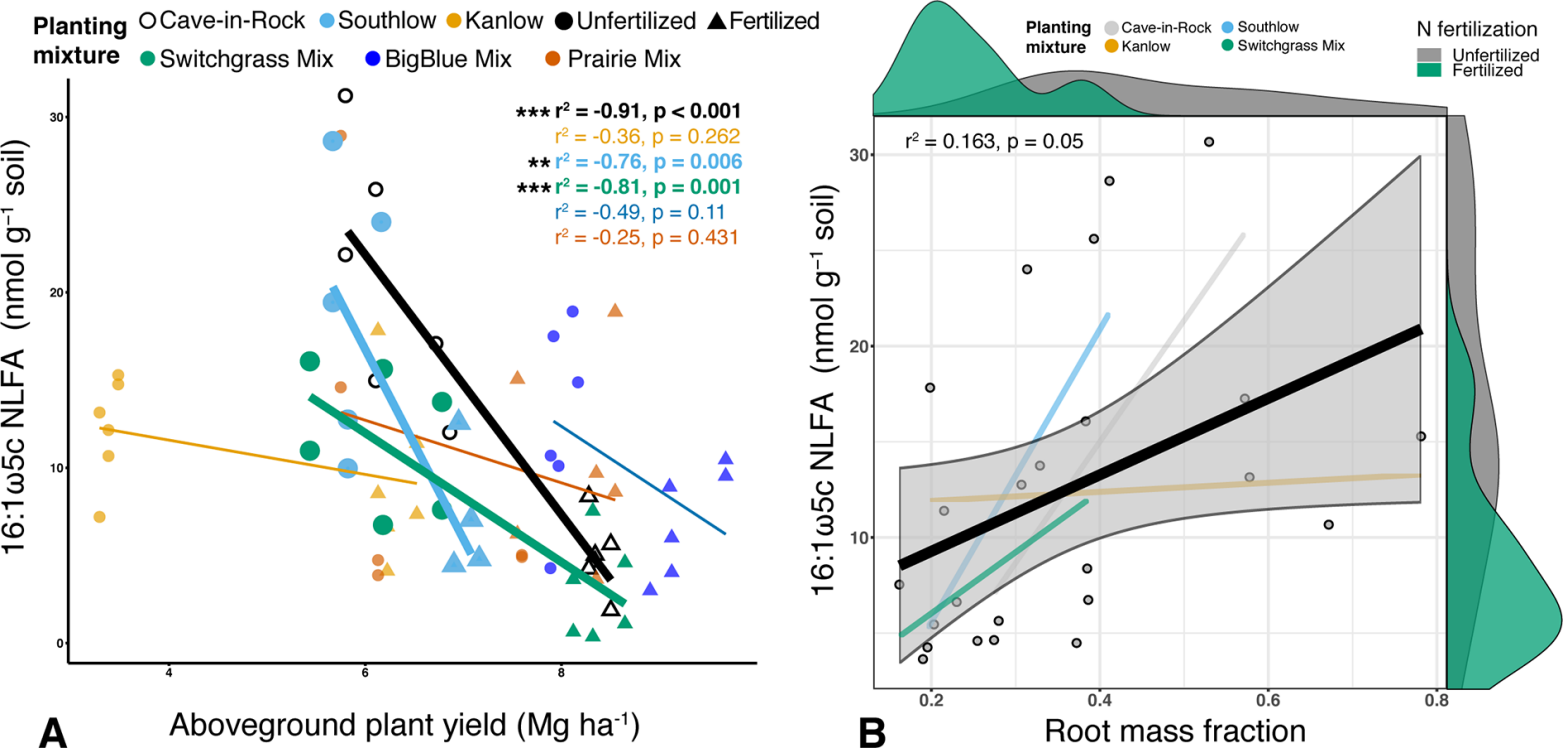
Relationship between AM fungal biomass in soil and aboveground plant biomass **(A)**, colored by planting mixture and either unfertilized (circles) or N-fertilized (triangles) plots. Cave-in-Rock (black/white), Southlow (light blue), and the switchgrass mixture plot (green) have significant negative correlations between aboveground plant biomass and the abundance of AM fungi in soils. Correlation statistics are provided in the upper right corner for all planting treatments. The relationship between AM fungal biomass in soil and root mass fraction **(B)** for all monocultures and the switchgrass mixture is represented by the black bar. 95% confidence limit is shaded, value densities for root mass fraction and NLFA abundance are on opposing x- and y-axes, and statistics are provided in the top left corner **p < 0.01, ***p ≤ 0.001.

**Figure 3:**
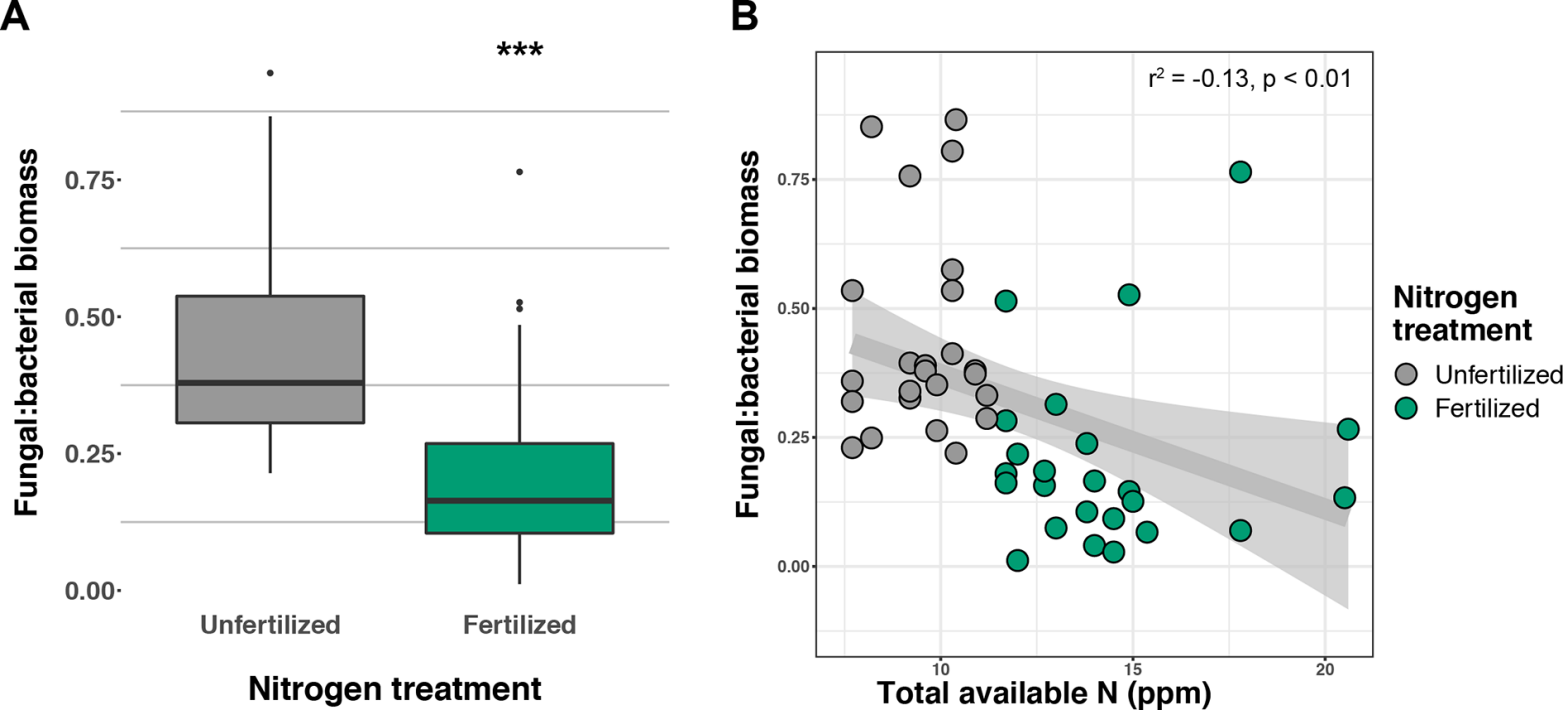
Response of fungal:bacterial biomass ratio to nitrogen (N) fertilization **(A)** and relationship of fungal:bacterial ratio to available soil inorganic N (NH^4+^ + NO^3-^) **(B)** ***p ≤ 0.001.

Soil concentration of the PLFA AM fungal biomarker (16:1ω5c) did not vary with +N or planting mixtures; however, NLFA decreased with +N (F_1,72_ = 19.37, p < 0.001; **Supplemental Figure 2**). There was a fertilization–by–planting mixture interaction (F_5,72_ = 3.413, p = 0.017; **Supplemental Figure 2**), with lower fungal biomass in +N Cave-in-Rock and Southlow moncultures (p < 0.005 and p = 0.02, respectively; **Supplemental Figure 2**). There were negative linear relationships between aboveground plant biomass yields (Mg ha^-1^) and fungal biomass for Cave-in-Rock (r^2^ = -0.91, p < 0.001; **Figure 2A**), Southlow (r^2^ = -0.76, p = 0.006), and the switchgrass mixture (r^2^ = -0.81, p = 0.001; **Figure 2A**). Root mass fraction was positively correlated with fungal biomass (r^2^ = 0.16, p = 0.05; **Figure 2B**). There was a fertilization–by–planting mixture interaction on total PLFA bacterial biomass (F_5,72_ = 3.56, p < 0.01); **Supplemental Figure 3**), and Tukey’s HSD revealed an increase in total bacterial PLFA biomass under the prairie mixture after +N (p < 0.05; **Supplemental Figure 3**). The F/B ratio was lower under +N than in unfertilized plots (F_1,72_ = 32.44, p < 0.001; **Figure 3A**), and there was a negative linear relationship with total available soil N (r^2^ = -0.16, p < 0.005; **Figure 3B**).

### Arbuscular Mycorrhizal Fungal Communities

**Figure 4:**
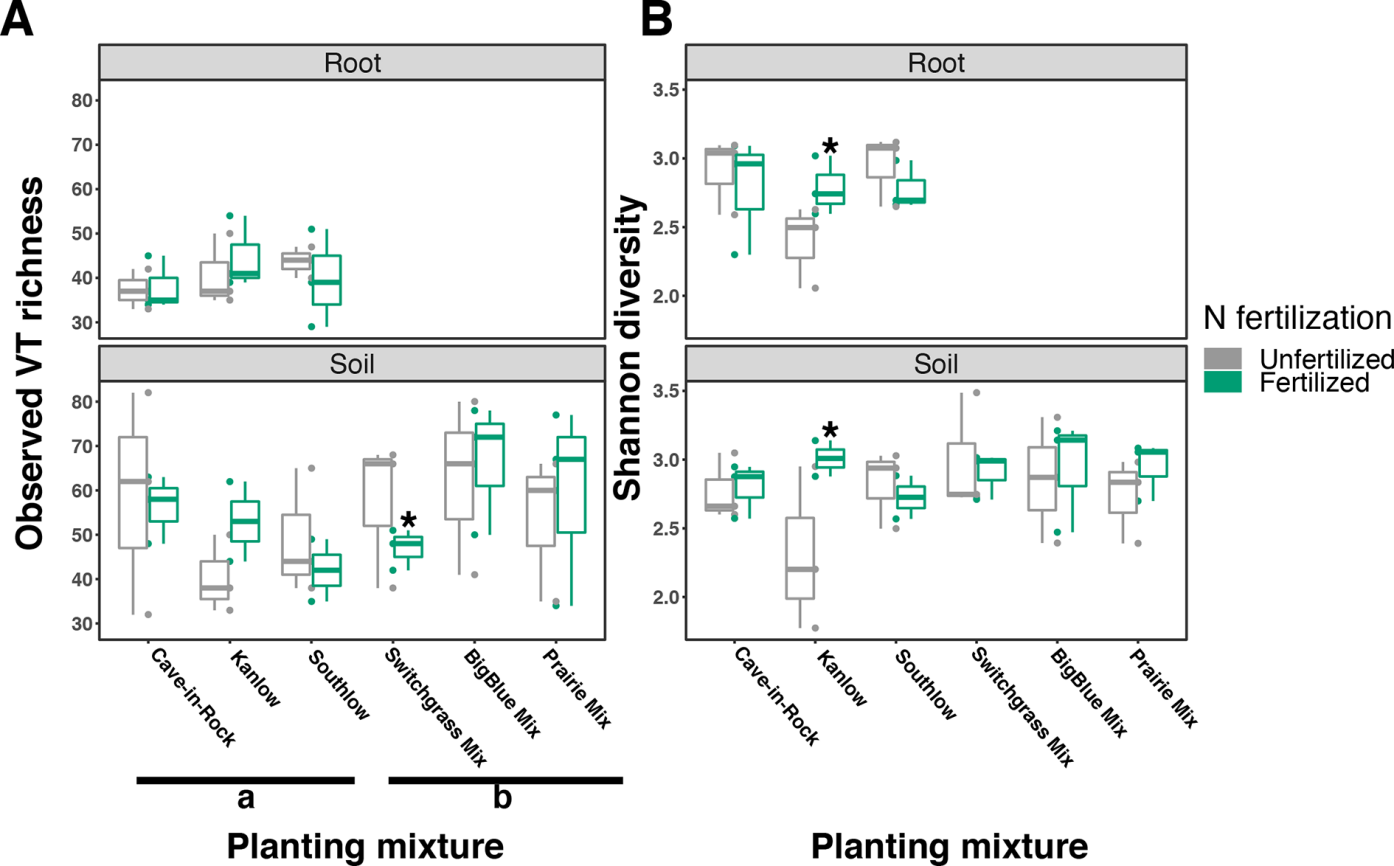
Arbuscular mycorrhizal (AM) fungal diversity observed in roots and soils measured as virtual taxa (VT) **(A)** and Shannon diversity **(B)** for all planting mixtures. Gray indicates unfertilized and green indicates N-fertilized treatments. Letters indicate significant differences between groups (adj-p < 0.05), and asterisks indicate significant ANOVA differences from fertilization within groups (p < 0.05).

**Figure 5:**
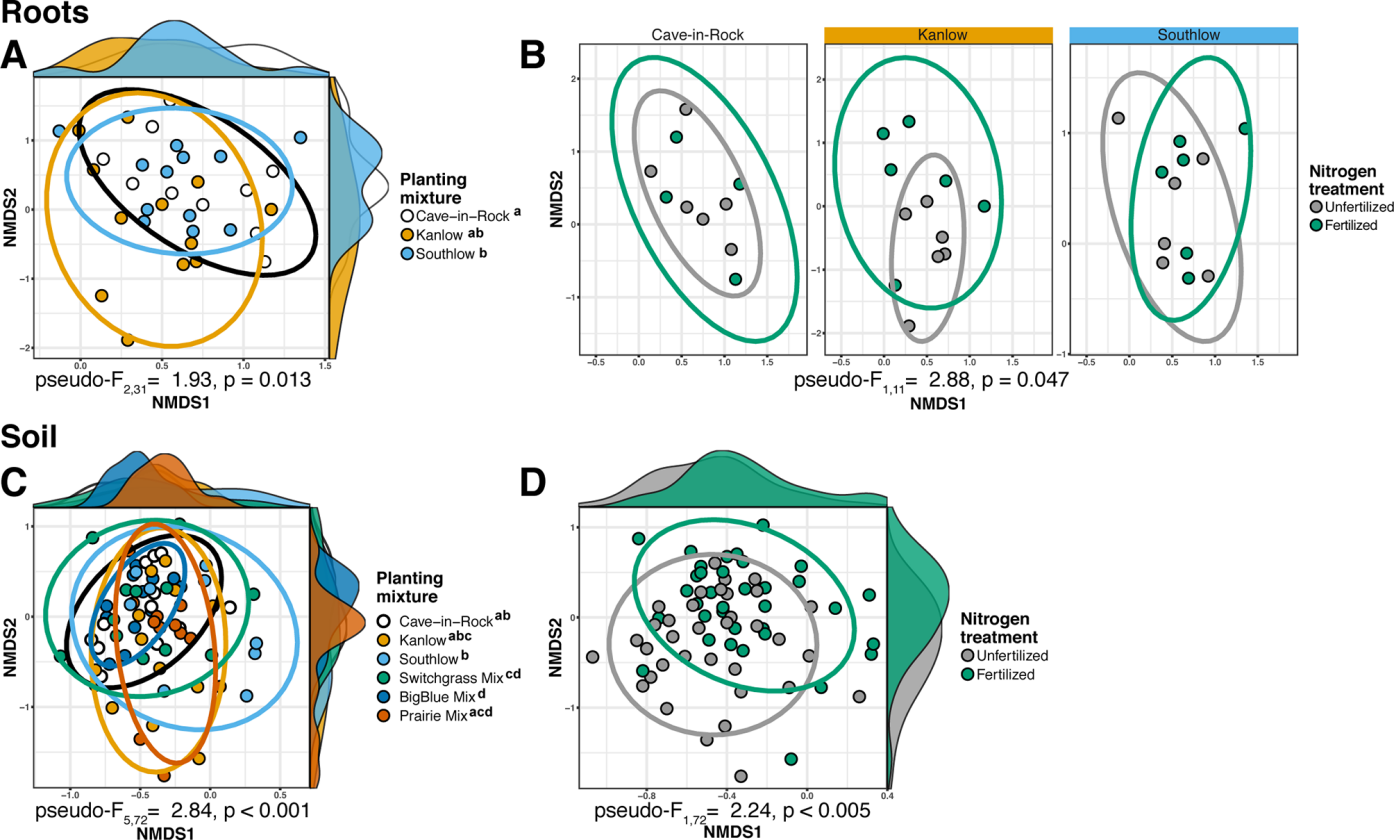
NMDS of AM fungal community Bray–Curtis dissimilarity from roots (top) and soils (bottom) colored by planting mixture **(A**, **C)** and colored by N fertilization treatment **(B**, **D)**. Ellipses represent 95% confidence areas around respective treatments. Letters indicate significant differences between groups (adj-p < 0.05).

The species richness of AM fungal communities in soil was higher for the combination of all interspecific diversity mixture plots than combined switchgrass monoculture plots (F_1,36_ = 3.77, p < 0.05; **Figure 4A**). Nitrogen fertilization reduced fungal richness in the soil of switchgrass mixture plots (adj-p < 0.01) and increased Shannon diversity (*H*) in roots and soils under Kanlow monocultures (p < 0.05 and p < 0.05, respectively; **Figure 4B**). The compositions of AM fungal communities in roots and soil were different (PERMANOVA Bray–Curtis; pseudo-F_1,108_ = 3.48, p < 0.001; **Supplemental Figure 4A**). The ß-diversity of AM fungal communities in roots was not altered by +N. Pairwise PERMANOVA indicated that the AM fungal community in roots of Cave-in-Rock was different from that in roots of Southlow (pseudo-F_2,31_ = 1.93, adj-p = 0.013; **Table 2**, **Figure 5A**), and +N influenced the Bray–Curtis dissimilarity of AM fungal communities in roots of the Kanlow cultivar (pseudo-F_1,11_ = 2.88, p < 0.05; **Table 2**, **Figure 5B**). The ß-diversity of AM fungal communities from soils was altered by planting mixture (PERMANOVA; pseudo-F_5,72_ = 2.84, p < 0.001; **Figure 5C**) and +N (PERMANOVA; pseudo-F_1,72_ = 2.24, p < 0.005; **Figure 5D**). The ß-diversity of the AM community in soil was influenced by planting mixture (**Table 2**) but not +N within planting mixtures (**Supplemental Figure 5**).

**Table 2:** Significant Bray–Curtis dissimilarities of AM fungal communities between planting mixtures or N fertilization within planting mixture from roots and soils calculated using pairwise PERMANOVA.

	pseudo-F	r^2^	adj-p-value
**Roots**			
*Between planting mixtures*			
Cave-in-Rock vs. Southlow	1.87	0.083	0.013
*Within Kanlow*			
Unfertilized vs. fertilized	1.88	0.130	0.024
**Soil**			
*Between planting mixtures*			
Cave-in-Rock vs. switchgrass mix	2.84	0.110	0.012
Cave-in-Rock vs. big blue mix	3.06	0.122	0.001
Kanlow vs. switchgrass mix	1.69	0.072	0.006
Kanlow vs. big blue mix	1.66	0.069	0.015
Southlow vs. switchgrass mix	1.56	0.066	0.039
Southlow vs. big blue mix	3.11	0.130	0.001
Southlow vs. prairie mix	2.61	0.105	0.002

**Figure 6:**
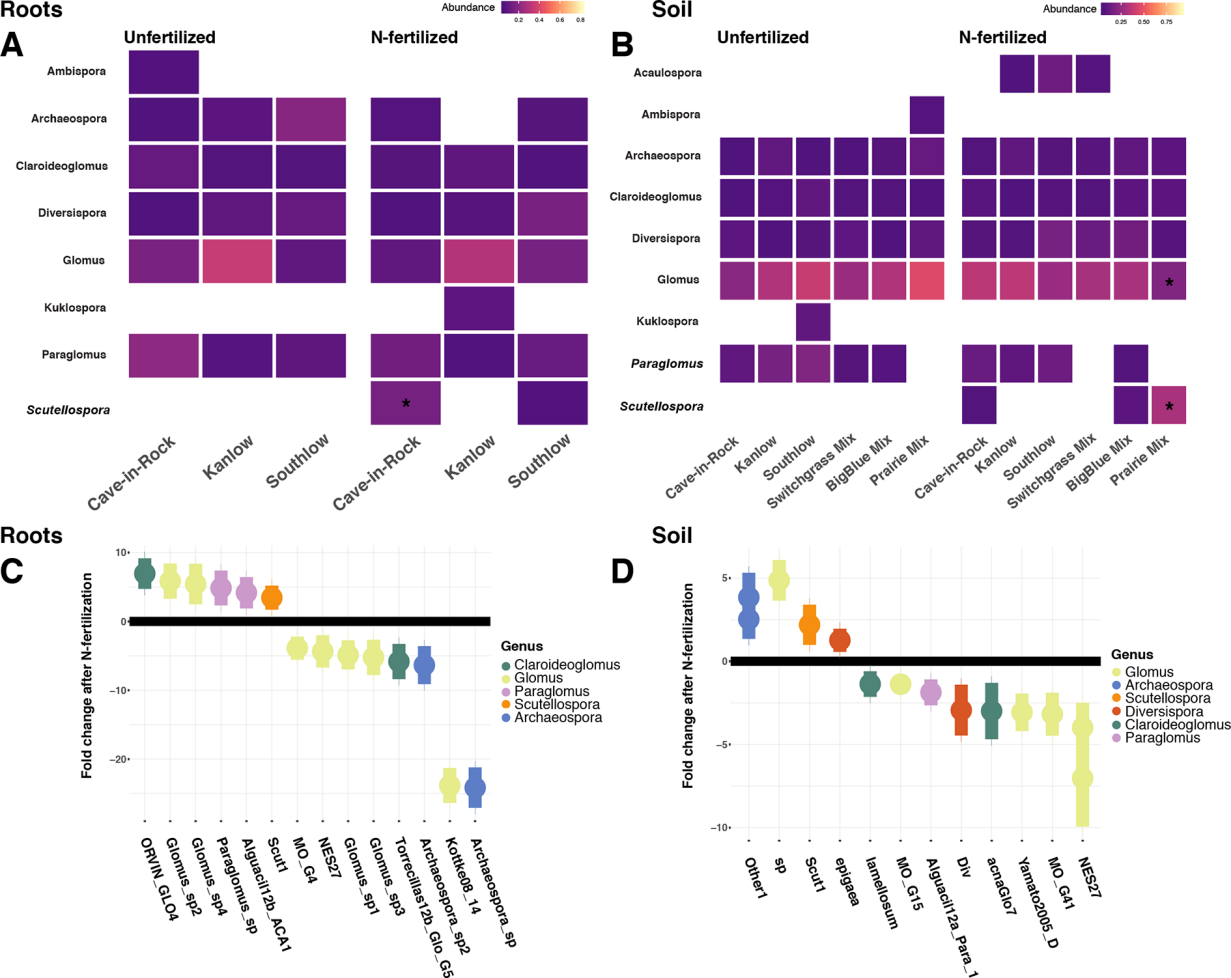
Heat maps of relative abundance of AM fungal genera for all planting treatments that are either unfertilized or N-fertilized, from roots **(A)** and soil **(B)**. The taxa presented here are core taxa, which represent only those genera present in more than 50% of all samples. Italicized taxa represent significant indicator taxa determined using either IndVal or r_pb_ (p < 0.05). Darker tiles in the heat map indicate lower relative abundance, and asterisks indicate a significant increase or decrease in relative abundance determined using Kruskal–Wallis H tests for each planting treatment after fertilization (FDR-p < 0.05). Empty tiles indicate that the taxa were not present in that treatment combination. The bottom **(C**–**D)** shows log_2_ fold change of AM fungal relative abundance that significantly differs after N fertilization (LRT; p < 0.05) for roots **(C)** and soils **(D)**. AM fungal “species” are on the x-axis, and points are colored by genus.

Relative abundance of core AM fungal taxa are presented as heat maps, and indicator species analyses revealed multiple indicator taxa for +N or certain planting mixture treatments (**Figures 6A–B**). The genus *Scutellospora* was an indicator under +N both in roots (IndVal = 0.81, fdr-p = 0.02; r_pb_ = 0.42, fdr-p = 0.02; **Figure 6A**) and in soils (IndVal = 0.65, fdr-p = 0.02; r_pb_ = 0.27, fdr-p = 0.058; **Figure 6B**). The genus *Gigaspora* was an indicator in soils under the switchgrass mixture (IndVal = 0.46, fdr-p = 0.036; r_pb_ = 0.39, fdr-p = 0.017), but it was not included in the heat map, because it was not observed in more than 50% of the samples. In soils under all three monocultures (Cave-in-Rock, Kanlow, Southlow), *Paraglomus* was determined to be an indicator genus (r_pb_ = 0.41, fdr-p = 0.01; **Figure 6B**). The relative abundance of *Scutellospora* was higher in +N plots (Kruskal–Wallis H = 11.72, FDR-p = 0.012; **Figures 6A–D**), and specifically in roots of Cave-in-Rock (H = 9.11, FDR-p < 0.01; **Figure 6A**), and in soil from prairie mixture plots (H = 14.25, FDR-p < 0.005; **Figure 6B**). *Glomus* was the most abundant genus across all treatments (**Supplemental Figure 6**), and the relative abundance of *Glomus* decreased in soil from prairie mixture +N plots (H = 13.6, FDR-p < 0.005; **Figure 6B**). Five AM fungal genera in roots and six genera in soils had significant log_2_ fold changes in relative abundance in +N plots (LRT; p < 0.05; **Figures 6C–D**). In roots, AM fungal taxa in the genera *Claroideoglomus* and *Glomus* had species that both increased and decreased with +N, while *Paraglomus* and *Scutellospora* increased with +N (**Figure 6C**). In soils, we found a similar pattern for *Glomus* and *Scutellospora* species, while *Arachaeospora* relative abundance increased and *Claroideoglomus* relative abundance declined with +N (**Figure 6D**).

### Bacterial Communities

**Figure 7:**
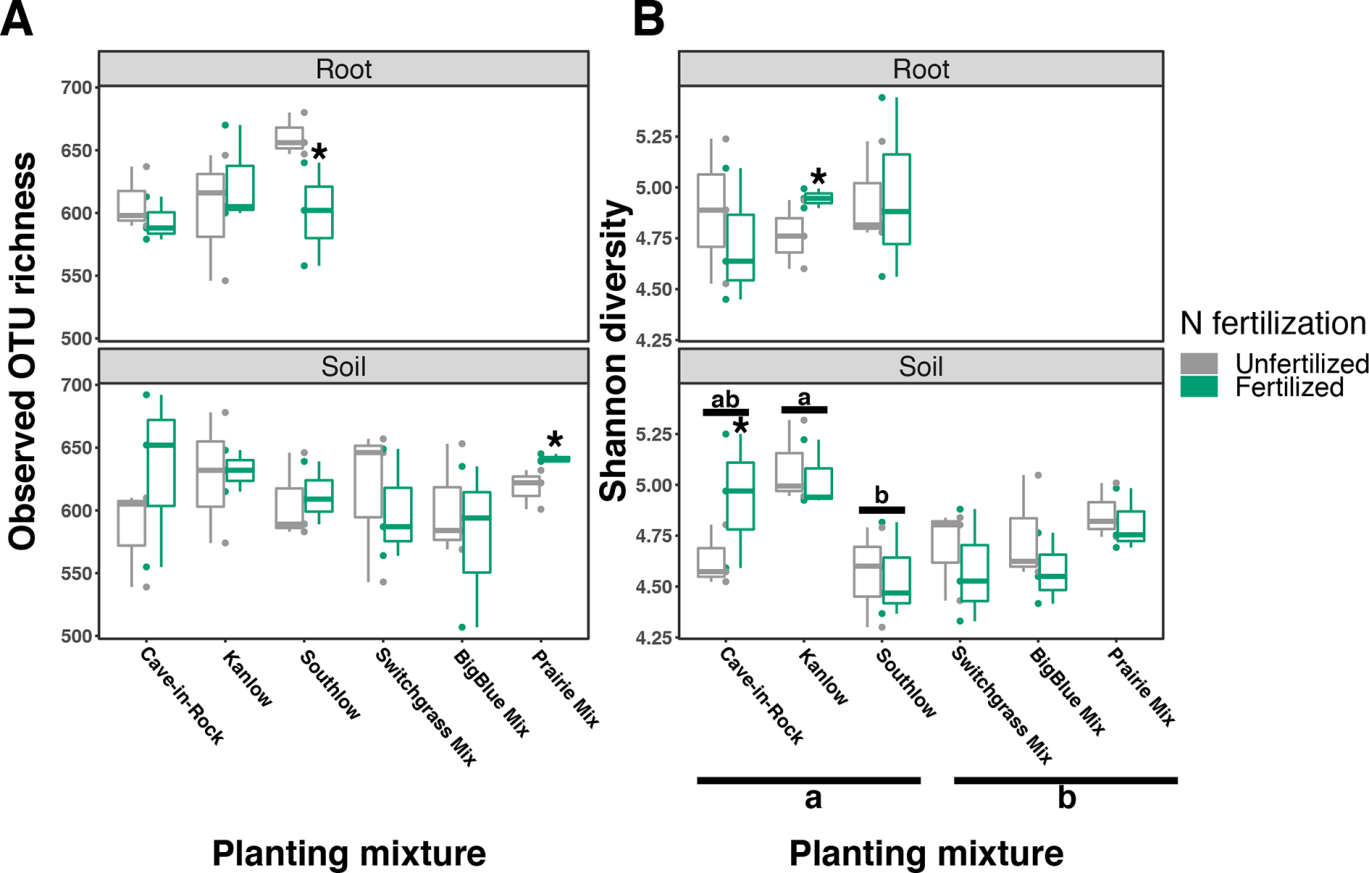
Bacterial diversity observed in roots and soils measured as OTU richness **(A)** and Shannon diversity **(B)** for all planting mixtures. Gray indicates unfertilized and green indicates N-fertilized treatments. Letters indicate significant differences between groups (adj-p < 0.05), and asterisks indicate significant ANOVA differences from fertilization within groups (p < 0.05).

**Figure 8:**
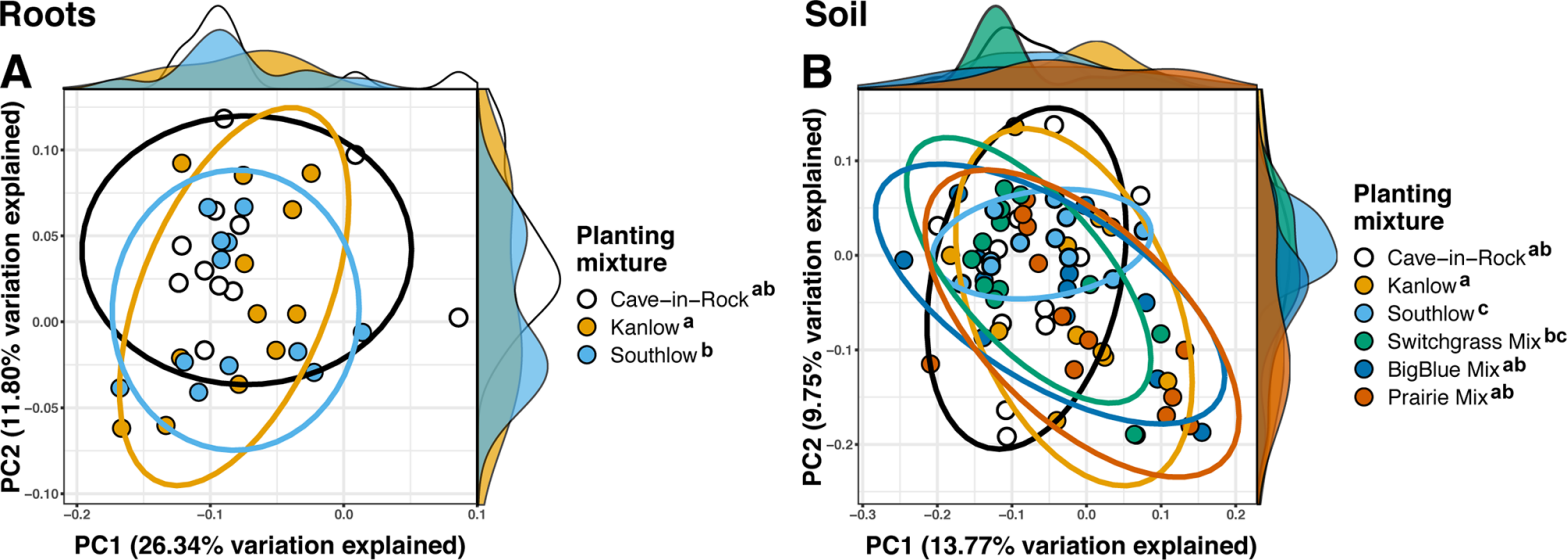
Principal coordinate analyses of bacterial community ß-diversity from roots **(A)**; weighted UniFrac or soils **(B)**; unweighted UniFrac, colored by planting treatment. Ellipses represent 95% confidence areas around respective treatments. Letters indicate significant differences between groups (adj-p < 0.05).

**Figure 9:**
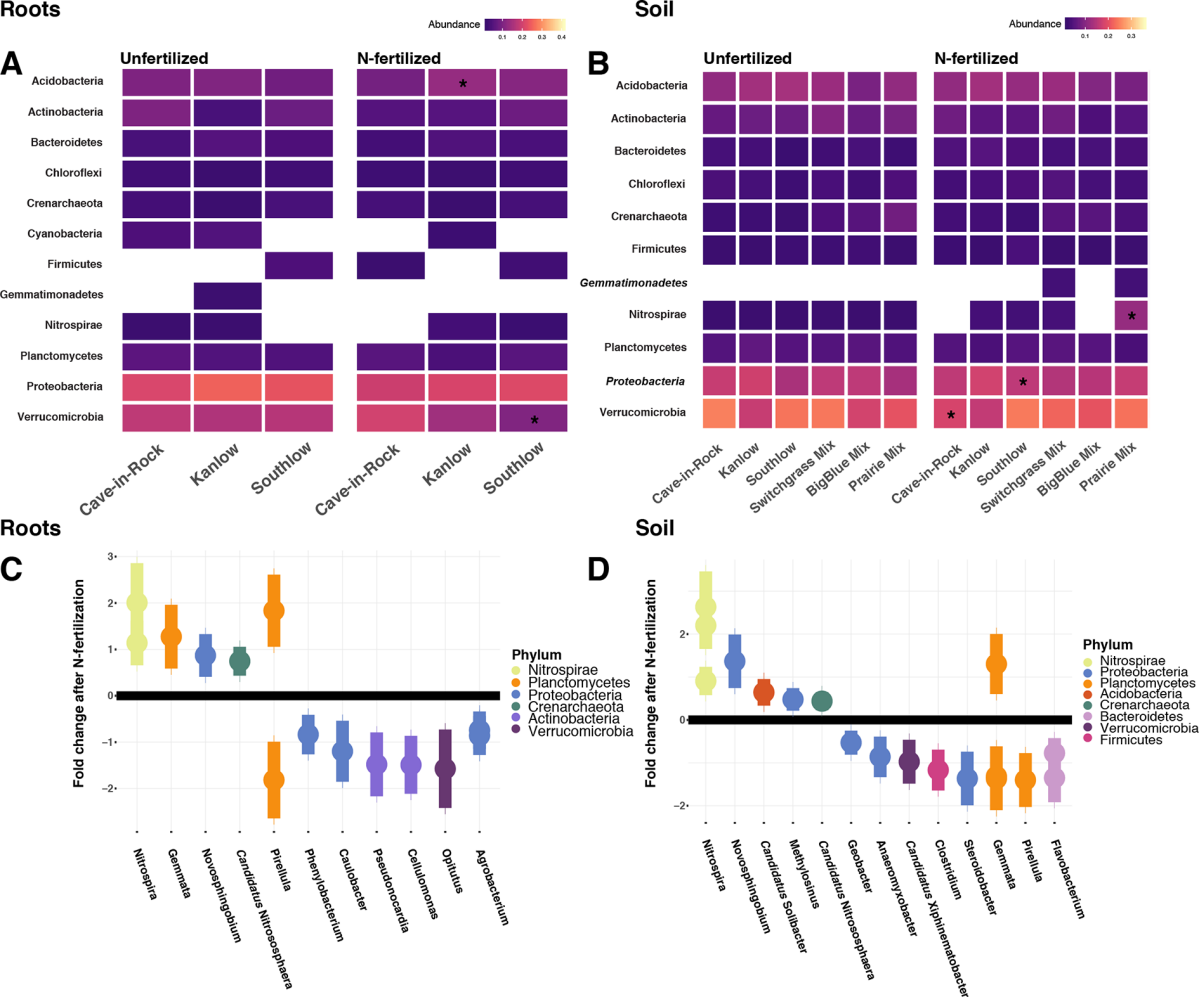
Heat maps of relative abundance of bacterial phyla for all planting treatments that are either unfertilized or N-fertilized, from roots **(A**, **C)** and soil **(B**, **D)**. The taxa presented here are core taxa, which represent only those phyla present in more than 50% of all samples. Italicized taxa represent significant indicator taxa determined using either IndVal or r_pb_ (p < 0.05). *Gemmatimonadetes* were indicator taxa for N-fertilized plots in soil **(B)**, while *Zetaproteobacteria* were indicator taxa for unfertilized plots in soil **(B)**. Darker-color tiles in the heat map indicate lower relative abundance, asterisks indicate a significant increase or decrease in relative abundance determined using Kruskal–Wallis H tests for each planting treatment (FDR-p < 0.05). Empty tiles indicate that the taxa were not present in that treatment combination. Log_2_ fold change of bacterial relative abundance that significantly differs after N fertilization (LRT; p < 0.05) for roots **(C)** and soils **(D)**. Bacterial families are on the x-axis, and points are colored by bacterial phylum.

Compared to other treatments, the richness of bacterial OTUs was lower on roots of Southlow in the +N plots (F_3,18_ = 2.8, p < 0.01; **Figure 7A**) and higher in soil under the prairie mixture (F_6,36_ = 3.8, p < 0.5; **Figure 7A**). Shannon diversity (*H*) of soil bacteria was higher in the combination of all monoculture plots compared to the combination of all diverse mixture plots (F_1,36_ = 2.1, p = 0.04; **Figure 7B**). Shannon diversity of soil bacteria in Kanlow plots was higher than in Southlow plots (F_2,18_ = 2.07, p < 0.01; **Figure 7B**). In +N plots, Shannon diversity of bacteria was higher on Kanlow roots and in soil in Cave-in-Rock plots (p < 0.05 and p < 0.05, respectively; **Figure 7B**). Bacterial communities were different in roots and soils (pseudo-F_1,108_ = 9.48, p < 0.001; **Supplemental Figure 4B**). Bacterial community weighted UniFrac ß-diversity from roots was altered by planting mixture (pseudo-F_2,35_ = 1.6, p = 0.012; **Figure 8A**). The bacterial community on roots of Southlow was different from Kanlow (pairwise PERMANOVA pseudo-F_1,12_ = 1.7, adj-p = 0.03; **Figure 8A**). Bacterial community unweighted and weighted UniFrac ß-diversity from soils were altered by planting mixture (pseudo-F_5,72_ = 1.34, p < 0.01, and pseudo-F_5,72_ = 1.54, p < 0.01, respectively; **Figure 8B**). The soil bacterial community under Southlow was different from all planting mixtures except for the switchgrass mixture (see **Table 3** for details, **Figure 8B**). Nitrogen fertilization had no effects on bacterial community ß-diversity from roots or soils; however, indicator species analyses of bacterial taxa revealed some differences. *Verrucomicrobia* and *Proteobacteria* were the most abundant phyla across all treatments (**Supplemental Figure 7**). The order N1423WL in the phylum *Gemmatimonadetes* was an indicator of +N in roots (IndVal = 0.44, fdr-p = 0.023), while in soils, RCP1-48 in the phylum *Gammaproteobacteria* was an indicator taxon of +N (IndVal = 0.58, fdr-p = 0.037). Bacterial order Mariprofundales (class *Zetaproteobacteria*) was an indicator taxa for unfertilized plots (IndVal = 0.5, fdr-p = 0.039). Bacteria in the phylum FCPU426 were indicator taxa associated with soils under Southlow monocultures (r_pb_ = 0.51, fdr-p = 0.026). *Acidobacteria* abundance increased on roots of Kanlow with +N (H = 9.7, p < 0.05; **Figure 9A**). The relative abundance of bacteria in the class *Alphaproteobacteria* was higher in soils under Southlow with +N (H = 7.04, FDR-p = 0.048; **Figure 9B**). *Nitrospirae* relative abundance was higher in soils under prairie mixtures with +N (H = 22.6, p < 0.01; **Figure 9B**). Verrucomicrobial relative abundance was lower on roots of Southlow with +N (H = 14.8, FDR-p < 0.01; **Figure 9A**) and lower in soils under Cave-in-Rock with +N (H = 9.4, FDR-p = 0.048; **Figure 9B**).

**Table 3:** Significant unweighted UniFrac differences of bacterial communities between planting mixtures from roots and soils calculated using pairwise PERMANOVA.

	pseudo-F	r^2^	adj-p-value
**Roots**			
*Between planting mixtures*			
Kanlow vs. Southlow	2.40	0.057	0.045
**Soil**			
*Between planting mixtures*			
Cave-in-Rock vs. Southlow	2.16	0.061	0.038
Kanlow vs. Southlow	2.72	0.074	0.015
Kanlow vs. switchgrass mix	3.30	0.089	0.011
Southlow vs. big blue mix	2.61	0.072	0.009
Southlow vs. prairie mix	2.29	0.063	0.027

Six bacterial phyla in roots and eight phyla in soils had significant log_2_ fold changes in relative abundance with +N (LRT; p < 0.05; **Figures 9C–D**). Relative abundance of *Verrucomicrobia* declined with +N under roots and soils (**Figures 9C–D**), as did *Actinobacteria* in roots (**Figure 9C**). *Nitrospirae* and *Crenarchaeota* consistently increased with +N, while taxa in the *Proteobacteria* and *Planctomycetes* exhibited both increases and decreases in relative abundance with +N (**Figures 9C–D**).

### NifH Gene Abundance

**Figure 10:**
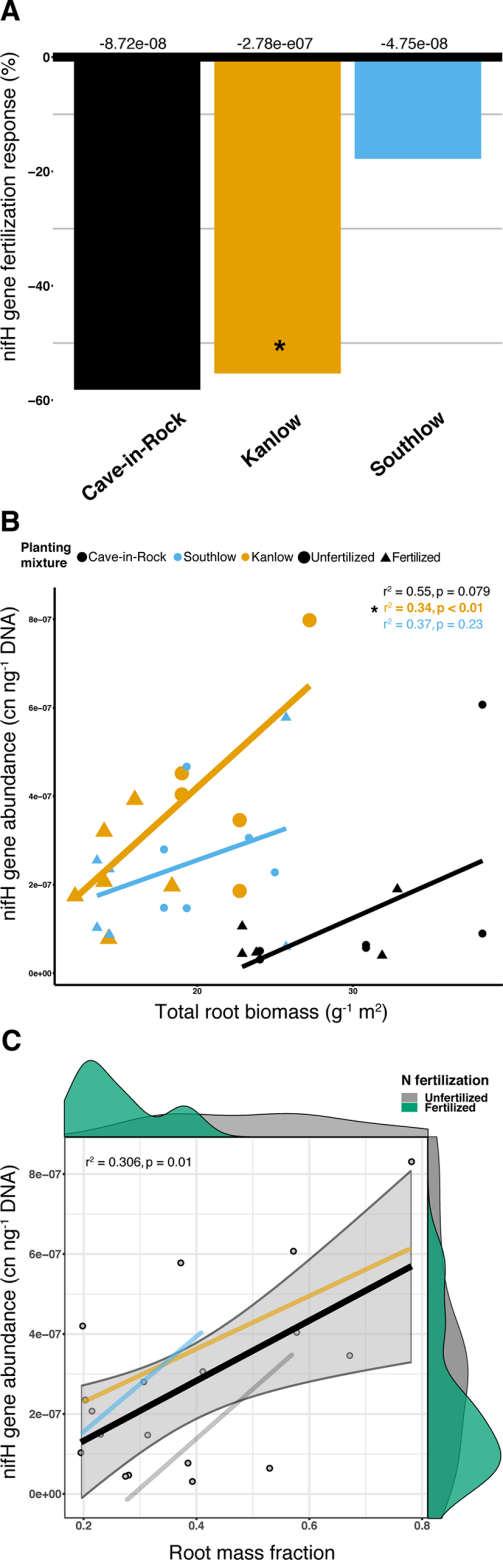
The abundance of nifH gene response (percent change) to N fertilization for three switchgrass monocultures **(A)**, where zero bar represents means from unfertilized plots. Values above the bars are mean decreases in total nifH gene abundance (copy number ng^-1^ DNA) per planting mixture. Relationship between nifH gene abundance and plant root biomass **(B)**, colored by planting mixture and either unfertilized (circles) or N-fertilized (triangles). Correlation statistics are provided in the upper right corner for each monoculture. The relationship between nifH gene abundance and root mass fraction **(C)** for all monocultures combined is represented by the black bar. 95% confidence limit is shaded, value densities for root mass fraction and nifH gene abundance colored by N fertilization treatment are on opposing x- and y-axes, and correlation statistic is provided in the top left corner.

Abundance of the nifH gene declined with +N (ANOVA; F_1,32_ = 5.16, p = 0.029; **Supplemental Figure 8**) and differed among switchgrass cultivars (ANOVA; F_2,32_ = 14.327, p < 0.001). Cave-in-Rock had the lowest total nifH gene abundance (p < 0.01; **Supplemental Figure 8**), and N fertilization decreased nifH abundance for Kanlow (F_1,10_ = 6.1, p < 0.05; **Figure 10A**). NifH gene abundance was positively correlated with root biomass under Kanlow (r^2^ = 034, p < 0.01; **Figure 10B**) and with root mass fraction across all monocultures (F_1,16_ = 8.25, adj-r^2^ = 0.306, p = 0.01; **Figure 10C**). There was no correlation between the relative abundance of *Verrucomicrobia* and nifH gene abundance from roots (**Supplemental Figure 9**).

## Discussion

Our results support the hypotheses that intra- and interspecific plant diversity influences soil microbial communities and that N fertilization (+N) shifts plant allocation of photosynthate towards aboveground production and away from root-associated microorganisms. The reduction in AM fungal biomass, nifH gene abundance, and F/B ratios after +N may reveal important functional trade-offs in particular planting treatments. Predictably, +N reduced the value of bacterial N-fixation (Kiers et al., 2003) as reflected in lower nifH gene abundance. Furthermore, the decrease in F/B ratio with +N might indicate a shift from nutrient conservation within mycorrhizal networks to a “leakier” system with more substrate available for opportunistic bacteria (de Vries et al., 2006; Fierer et al., 2009). The shift in allocation from belowground nutritional symbioses to aboveground plant structures provides support for optimal resource allocation strategies in the extended plant phenotype, particularly when adopting the collective view of functions and interactions among plants and microbial consortia (Johnson et al., 2003a; Vandenkoornhuyse et al., 2015).

### Plant Biomass, Nutritional Symbioses, and Plant Diversity

Aboveground plant growth increased after +N for all planting treatments, but the strength of these responses was dependent on planting mixture (**Figure 1**). For example, the big bluestem mixture produced the greatest aboveground biomass of all the planting treatments (**Figure 1A**), but +N only increased biomass by ∼17%. Kanlow monocultures, on the other hand, had the lowest total yields, and +N increased aboveground biomass by ∼85% (**Figure 1**). Further, Kanlow allocated significantly more biomass aboveground to shoots after +N but did not significantly decrease allocation to AM fungal biomass in soil (**Figures 1C**, **2A**), despite it being the only switchgrass cultivar to exhibit significant changes in AM fungal communities in response to +N (**Figure 5B**). Compared to the other cultivars, unfertilized Kanlow monocultures had higher total abundance of nifH in the rhizosphere that was positively correlated with root biomass (**Supplemental Figure 7**; **Figure 10**). Furthermore, Kanlow was the only cultivar in which +N caused a significant decrease in root mass fraction (**Supplemental Figure 1B**). The “case study” of Kanlow might suggest that this switchgrass cultivar reduces its reliance on bacterial N-fixing activity and selectively utilizes particular AM fungal taxa to acquire co-limiting resources in soil (e.g., P), although this was not specifically tested and warrants further study.

In contrast to the monoculture treatments, the big bluestem and prairie mixtures maintained high aboveground productivity and responded less strongly to +N. In the prairie mixture, there was no reduction in AM fungal biomass in soil with +N (**Supplemental Figure 2**). NifH gene abundance was not measured in roots collected from the diverse planting mixtures, but given the lower growth response to +N, high aboveground productivity, and maintenance of C allocation to AM fungal biomass, it would be interesting to determine if N-fixation under the more diverse big bluestem mixture is maintained or even increased with +N, as was observed in a long-term field experiment in Minnesota (Revillini, 2018). Overall, our study indicates that functional responses to +N depended on plant intra- or interspecific diversity. These cultivar-dependent shifts can be explained by a variety of mechanisms, but our data may indicate selective shifts in microbial associations.

### The AM fungal Microbiome

Our findings do not support the hypothesis that species richness of AM fungi should be higher with diverse plant communities compared to monocultures. Responses of AM fungal richness and diversity to plant diversity and +N were subtle and highly variable across the treatments (**Figure 4**). The ß-diversity of AM fungal communities also responded variably to planting mixture whether in roots or soils (**Table 2**) and to +N across plant diversity treatments (**Figure 5B**). Indicator analyses and significant changes in relative abundances of particular AM fungal taxa suggest that AM fungal taxa are individualistic in their responses to plant diversity and N availability (**Figures 6**). For example, *Scutellospora* increased in relative abundance with +N, in roots of Cave-in-Rock monocultures, and in +N prairie mixture plots (**Figure 6**). Fungi within the genus *Scutellospora* have been shown to both increase and decrease in response to N enrichment depending on the study (Johnson et al., 1992; Johnson, 1993; Egerton-Warburton et al., 2007). Environmental context, especially the relative availability of limiting resources, influences the structure and function of AM symbioses (Hoeksema et al., 2010; Johnson, 2010). We observed that plant diversity and +N influenced the structure of AM fungal communities, and additional research is necessary to determine specific functional relationships between switchgrass cultivars and AM fungal taxa. Previous studies have measured the performance of *individual* plant–AM fungal taxa combinations (Ji and Bever, 2016), as well as the *cumulative effects of entire suites* of AM fungi in soils (Bennett and Klironomos, 2018; Kulmatiski, 2018). Substantially more research must be performed in field-based studies to sufficiently elucidate the dynamic interactions among plants, AM fungi, and other soil communities before the mechanisms by which plants engage and select for soil biota to enhance nutrient availability can be understood.

### The Bacterial Microbiome

As with AM fungi, plant diversity was not a good predictor of bacterial α-diversity. This supports the conclusion that plant and bacterial diversity are often uncoupled (Wardle, 2006; Prober et al., 2015). However, the ß-diversity of bacterial communities in roots and soils was influenced by the diversity and composition of plants (**Table 3**, **Figure 8**). Bacterial indicator taxa and shifts in relative abundance under +N were observed under the different planting combinations (**Figure 9**). Relative abundance of *Verrucomicrobia* declined significantly with +N (**Figures 9C–D**), on roots of Southlow (**Figure 9A**), and in soils under Cave-in-Rock (**Figure 9B**). This finding corroborates previous studies that show that verrucomicrobial abundance is influenced by N availability in soils (Ramirez et al., 2012; Shen et al., 2017). *Gemmatimonadetes* were indicators of +N in our study, which supports results from Nemergut et al. (2008). It has been proposed that bacterial taxa dominant and active in the rhizosphere, including *Gemmatimonadetes*, may be superior competitors for plant-derived C exudates (Gkarmiri et al., 2017), which can increase under +N despite reduced root biomass allocation (Badri and Vivanco, 2009).

Increasing resource availability can influence the abundance and functioning of bacterial taxa involved with nutrient cycling (Dai et al., 2018). Relative abundance of bacterial taxa related to N-cycling declined predictably after +N in our experiment. Putative N_2_-fixing taxa determined previously from switchgrass roots (Bahulikar et al., 2014) such as *Rhizobium*, *Methylobacterium*, *Burkholderia*, and *Azoarcus* were present in roots and soil in our experiment. As predicted, the relative abundance of the putative N_2_-fixing families *Agrobacterium*, *Caulobacter*, and *Clostridium* (Zehr et al., 2003; Bahulikar et al., 2014) declined significantly after +N (**Figures 9C–D**). Known nitrifying taxa, *Nitrospira* and *Crenarchaeota* consistently increased in relative abundance after +N (**Figures 9C–D**), which supports conclusions of many previous studies that these taxa should become more abundant with N enrichment (Treusch et al., 2005; Zhou et al., 2015). Interestingly, a previous study showed a potential competitive shift between bacterial *Nitrospira* and archaeal *Crenarchaeota* at high N fertilization rates, but this was not observed in our experiment (Bates et al., 2011). Additional research is necessary to examine how shifts in the structure of bacterial communities may influence their function on roots and in the soil.

## Conclusions

Our results provide a baseline for switchgrass microbiome research. Functional diversity of soil microbial symbionts, though sometimes ecologically coherent and exhibiting phylogenetic trait conservatism (Martiny et al., 2013; Philippot et al., 2010), is influenced by taxa-specific interactions with the environment and other organisms (Martiny et al., 2015). We focused on the important *nutritional* relationships between plants and their AM fungal or bacterial microbiomes in the context of resource trade theories but are aware of other valuable functional roles played by both AM fungal and rhizobacterial communities such as biological control of pathogens and pests, drought tolerance, or induced systemic resistance. Research and methods that can further tease apart the multifunctionality of microbiomes in field studies will be critical to the developing field of plant and soil microbiome management in grasslands (Bender et al., 2016; Toju et al., 2018). This work can help generate hypotheses for future targeted studies that link microbiome assembly with microbial function and facilitation of plant nutrition and health. For example, what relationships can be identified between plant nutrition, plant productivity, or bioenergy production and the shifts in microbial nutrient cycling functions? How are quantities and types of soil organic carbon under different planting mixtures affecting bacterial community structure and function in perennial feedstock cropping systems over time? Across broad geographic ranges, are there consistent plant–microbial responses to fertilization regimes? Future research of plant growth–promoting microorganisms should identify specific P- and N-cycling microbial functions in field-based studies along nutrient availability gradients to determine thresholds where microbial benefits to plants can be maximized.

## Data Availability

The raw data supporting the conclusions of this manuscript will be made available by the authors, without undue reservation, to any qualified researcher.

## Author Contribution

DR, NJ, GW, and MM conceived of the experiment. DR performed the majority of the sampling, analyses, and writing. RL performed qPCR of the nifH gene, and all authors contributed equally to manuscript preparation.

## Funding

Funding was provided by the USDA-AFRI (2010-03894) to GW, MM, and NJ, an NAU Genes to Environment Fellowship to DR, and also science-supporting friends and family of DR on the crowdsourcing site experiment.com (doi:10.18258/7891).

## Conflict of Interest Statement

The authors declare that the research was conducted in the absence of any commercial or financial relationships that could be constructed as a potential conflict of interest.

## References

[B1] Adesemoye A. O. Torbert H. A. Kloepper J. W. (2009). Plant growth–promoting rhizobacteria allow reduced application rates of chemical fertilizers. Microb. Ecol. 58, 921–929. 10.1007/s00248-009-9531-y 19466478

[B2] Adkins J. Jastrow J. D. Morris G. P. Six J. de Graaff M.-A. (2016). Effects of switchgrass cultivars and intraspecific differences in root structure on soil carbon inputs and accumulation. Geoderma 262, 147–154. 10.1016/j.geoderma.2015.08.019

[B3] Agler M. T. Ruhe J. Kroll S. Morhenn C. Kim S. Weigel D. (2016). Microbial hub taxa link host and abiotic factors to plant microbiome variation. PLoS Biol. 14, e1002352. 10.1371/journal.pbio.1002352 26788878 PMC4720289

[B4] Alori E. T. Glick B. R. Babalola O. O. (2017). Microbial phosphorus solubilization and its potential for use in sustainable agriculture. Front. Microbiol. 8, 1–8. 10.3389/fmicb.2017.00971 28626450 PMC5454063

[B5] Anderson M. J. (2001). A new method for non-parametric multivariate analysis of variance. Austral. Ecol. 26, 32–46. 10.1111/j.1442-9993.2001.tb00081.x

[B6] Azcón-Aguilar C. Barea J. M. (2015). Nutrient cycling in the mycorrhizosphere. J. Soil Sci. Plant Nutr. 25, 372–396. 10.4067/S0718-95162015005000035

[B7] Badri D. V. Vivanco J. M. (2009). Regulation and function of root exudates. Plant. Cell Environ. 32, 666–681. 10.1111/j.1365-3040.2009.01926.x 19143988

[B8] Bahulikar R. A. Torres-Jerez I. Worley E. Craven K. Udvardi M. K. (2014). Diversity of nitrogen-fixing bacteria associated with switchgrass in the native tallgrass prairie of Northern Oklahoma. Appl. Environ. Microbiol. 80, 5636–5643. 10.1128/AEM.02091-14 25002418 PMC4178587

[B9] Bakker P. A. H. M. Pieterse C. M. J. de Jonge R. Berendsen R. L. (2018). The soil-borne legacy. Cell 172, 1178–1180. 10.1016/j.cell.2018.02.024 29522740

[B10] Bardgett R. D. van der Putten W. H. (2014). Belowground biodiversity and ecosystem functioning. Nature 515, 505–511. 10.1038/nature13855 25428498

[B11] Bates S. T. Berg-Lyons D. Caporaso J. G. Walters W. Knight R. Fierer N. (2011). Examining the global distribution of dominant archaeal populations in soil. ISME J. 5, 908–917. 10.1038/ismej.2010.171 21085198 PMC3105767

[B12] Bauer J. T. Kleczewski N. M. Reynolds H. L. Bever J. D. Clay K. (2012). Nitrogen-fixing bacteria, arbuscular mycorrhizal fungi, and the productivity and structure of prairie grassland communities. Oecologia 170, 1089–1098. 10.1007/s00442-012-2363-3 22684866

[B13] Bender S. F. Wagg C. van der Heijden M. G. A. (2016). An underground revolution: biodiversity and soil ecological engineering for agricultural sustainability. Trends Ecol. Evol. 31, 440–452. 10.1016/j.tree.2016.02.016 26993667

[B14] Bennett J. A. Klironomos J. (2018). Mechanisms of plant–soil feedback: interactions among biotic and abiotic drivers. New Phytol. 91–96. 10.1002/ecs2.2132 30451287

[B15] Berg G. Grube M. Schloter M. Smalla K. (2014). Unraveling the plant microbiome: Looking back and future perspectives. Front. Microbiol. 5, 1–7. 10.3389/fmicb.2014.00148 24926286 PMC4045152

[B16] Bergmann G. T. Bates S. T. Eilers K. G. Lauber C. L. Caporaso J. G. Walters W. A. (2012). The under-recognized dominance of Verrucomicrobia in soil bacterial communities. Soil Biol. Biochem. 43, 1450–1455. 10.1016/j.soilbio.2011.03.012 PMC3260529 22267877

[B17] Bever J. D. (2015). Preferential allocation, physio-evolutionary feedbacks, and the stability and environmental patterns of mutualism between plants and their root symbionts. New Phytol. 205, 1503–1514. 10.1111/nph.13239 25561086

[B18] Bever J. D. Richardson S. C. Lawrence B. M. Holmes J. Watson M. (2009). Preferential allocation to beneficial symbiont with spatial structure maintains mycorrhizal mutualism. Ecol. Lett. 12, 13–21. 10.1111/j.1461-0248.2008.01254.x 19019195

[B19] Bokulich N. Subramanian S. Faith J. J. Gevers D. Gordon J. I. Knight R. (2012). Quality-filtering vastly improves diversity estimates from Illumina amplicon sequencing. Nat. Methods 10, 57–59. 10.1038/nmeth.2276 23202435 PMC3531572

[B20] Bouton J. H. (2007). Molecular breeding of switchgrass for use as a biofuel crop. Curr. Opin. Genet. Dev. 17, 553–558. 10.1016/j.gde.2007.08.012 17933511

[B21] Brejda J. J. Moser L. E. Vogel K. P. (1998). Evaluation of switchgrass rhizosphere microflora for enhancing seedling yield and nutrient uptake. Agron. J. 90, 753–758. 10.2134/agronj1998.00021962009000060006x

[B22] Burger M. Jackson L. E. (2003). Microbial immobilization of ammonium and nitrate in relation to ammonification and nitrification rates in organic and conventional cropping systems. Soil Biol. Biochem. 35, 29–36. 10.1016/S0038-0717(02)00233-X

[B23] Busby P. E. Soman C. Wagner M. R. Friesen M. L. Kremer J. Bennett A. (2017). Research priorities for harnessing plant microbiomes in sustainable agriculture. PLoS Biol. 15, 1–14. 10.1371/journal.pbio.2001793 PMC5370116 28350798

[B24] Caporaso J. G. Bittinger K. Bushman F. D. Desantis T. Z. Andersen G. L. Knight R. (2010a). PyNAST: a flexible tool for aligning sequences to a template alignment. Bioinformatics 26, 266–267. 10.1093/bioinformatics/btp636 19914921 PMC2804299

[B25] Caporaso J. G. Kuczynski J. Stombaugh J. Bittinger K. Bushman F. D. Costello E. K. (2010b). QIIME allows analysis of high-throughput community sequencing data. Nat. Methods 7, 335–336. 10.1038/nmeth.f.303 20383131 PMC3156573

[B26] Chagnon P. L. Bradley R. L. (2013). Evidence that soil nutrient stoichiometry controls the competitive abilities of arbuscular mycorrhizal vs. root-borne non-mycorrhizal fungi. Fungal Ecol. 6, 557–560. 10.1016/j.funeco.2013.09.005

[B27] Dai Z. Su W. Chen H. Barberán A. Zhao H. Yu M. (2018). Long-term nitrogen fertilization decreases bacterial diversity and favors the growth of Actinobacteria and Proteobacteria in agro-ecosystems across the globe. Glob. Chang. Biol. 24, 3452–3461. 10.1111/gcb.14163 29645398

[B28] De Cáceres M. Legendre P. (2009). Associations between species and groups of sites: indices and statistical inference. Ecology 90, 3566–3574. 10.1890/08-1823.1 20120823

[B29] De Cáceres M. Legendre P. Moretti M. (2010). Improving indicator species analysis by combining groups of sites. Oikos 119, 1674–1684. 10.1111/j.1600-0706.2010.18334.x

[B30] de Vries F. T. Hoffland E. van Eekeren N. Brussaard L. Bloem J. (2006). Fungal/bacterial ratios in grasslands with contrasting nitrogen management. Soil Biol. Biochem. 38, 2092–2103. 10.1016/j.soilbio.2006.01.008

[B31] Dumbrell A. J. Ashton P. D. Aziz N. Feng G. Nelson M. Dytham C. (2011). Distinct seasonal assemblages of arbuscular mycorrhizal fungi revealed by massively parallel pyrosequencing. New Phytol. 190, 794–804. 10.1111/j.1469-8137.2010.03636.x 21294738

[B32] Egerton-Warburton L. M. Johnson N. C. Allen E. B. (2007). Mycorrhizal community dynamics following nitrogen fertilization: a cross-site test in five grasslands. Ecol. Monogr. 77, 527–544. 10.1890/06-1772.1

[B33] Fierer N. Ladau J. Clemente J. C. Leff J. W. Owens S. M. Pollard K. S. (2013). Reconstructing the microbial diversity and function of pre-agricultural tallgrass prairie soils in the United States. Science 342, 621–624. 10.1126/science.1243768 24179225

[B34] Fierer N. Strickland M. S. Liptzin D. Bradford M. A. Cleveland C. C. (2009). Global patterns in belowground communities. Ecol. Lett. 12, 1238–1249. 10.1111/j.1461-0248.2009.01360.x 19674041

[B35] Frostegård A. Bååth E. (1996). The use of phospholipid fatty acid analysis to estimate bacterial and fungal biomass in soil. Biol. Fertil. Soils 22, 59–65. 10.1007/s003740050076

[B36] Frostegård Å. Tunlid A. Bååth E. (2011). Use and misuse of PLFA measurements in soils. Soil Biol. Biochem. 43, 1621–1625. 10.1016/j.soilbio.2010.11.021

[B37] Gaby J. C. Buckley D. H. (2012). A comprehensive evaluation of PCR primers to amplify the nifH gene of nitrogenase. PLoS ONE. 7, e42149. 22848735 10.1371/journal.pone.0042149 PMC3405036

[B38] Gilbert J. A. Blaser M. J. Caporaso J. G. Jansson J. K. Lynch S. V. Knight R. (2018). Current understanding of the human microbiome. Nat. Med. 24, 392. 10.1038/nm.4517 29634682 PMC7043356

[B39] Gilbert J. A. Jansson J. K. Knight R. (2014). The Earth Microbiome Project: successes and aspirations. BMC Biol. 12, 69. 10.1186/s12915-014-0069-1 25184604 PMC4141107

[B40] Gkarmiri K. Mahmood S. Ekblad A. Finlay R. (2017). Identifying the active microbiome associated with roots and rhizosphere soil of oilseed rape. Appl. Environ. Microbiol. 83, 1–14. 10.1128/AEM.01938-17 PMC5666129 28887416

[B41] Govindarajulu M. Pfeffer P. E. Jin H. Abubaker J. Douds D. D. Allen J. W. (2005). Nitrogen transfer in the arbuscular mycorrhizal symbiosis. Nature 435, 819–823. 10.1038/nature03610 15944705

[B42] Gray S. B. Classen A. T. Kardol P. Yermakov Z. Michael Miller R. (2011). Multiple climate change factors interact to alter soil microbial community structure in an old-field ecosystem. Soil Sci. Soc. Am. J. 75, 2217. 10.2136/sssaj2011.0135

[B43] Hannula S. E. Morriën E. De Hollander M. Van Der Putten W. H. Van Veen J. A. De Boer W., (2017). Shifts in rhizosphere fungal community during secondary succession following abandonment from agriculture. ISME J. 11 (10), 2294–2304. 10.1038/ismej.2017.90 28585935 PMC5607372

[B44] Hart M. M. Trevors J. T. (2005). Microbe management: application of mycorrhizal fungi in sustainable agriculture. Front. Ecol. Environ. 3, 533–539. 10.1890/1540-9295(2005)003[0533:MMAOMF]2.0.CO;2

[B45] Hodge A. Storer K. (2015). Arbuscular mycorrhiza and nitrogen: implications for individual plants through to ecosystems. Plant Soil. 386 (1–2), 1–19. 10.1007/s11104-014-2162-1

[B46] Hoeksema J. D. Chaudhary V. B. Gehring C. A. Johnson N. C. Karst J. Koide R. T. (2010). A meta-analysis of context-dependency in plant response to inoculation with mycorrhizal fungi. Ecol. Lett. 13, 394–407. 10.1111/j.1461-0248.2009.01430.x 20100237

[B47] Hungate B. A. Barbier E. B. Ando A. W. Marks S. P. Reich P. B. Gestel N. (2017). The economic value of grassland species for carbon storage. Sci. Adv. 3 (4), e1601880. 10.1126/sciadv.1601880 28435876 PMC5381958

[B48] Jach-Smith L. C. Jackson R. D. (2018). N addition undermines N supplied by arbuscular mycorrhizal fungi to native perennial grasses. Soil Biol. Biochem. 116, 148–157. 10.1016/j.soilbio.2017.10.009

[B49] Ji B. Bever J. D. (2016). Plant preferential allocation and fungal reward decline with soil phosphorus: implications for mycorrhizal mutualism. Ecosphere 7, 1–11. 10.1002/ecs2.1256

[B50] Johansson J. F. Paul L. R. Finlay R. D. (2004). Microbial interactions in the mycorrhizosphere and their significance for sustainable agriculture. FEMS Microbiol. Ecol. 48, 1–13. 10.1016/j.femsec.2003.11.012 19712426

[B51] Johnson N. C. (1993). Can fertilization of soil select less mutualistic mycorrhizae? Ecol. Appl. 3, 749–757. 10.2307/1942106 27759303

[B52] Johnson N. C. (2010). Resource stoichiometry elucidates the structure and function of arbuscular mycorrhizas across scales. New Phytol. 185, 631–647. 10.1111/j.1469-8137.2009.03110.x 19968797

[B53] Johnson N. C. Rowland D. L. Corkidi L. Egerton-Warburton L. M. Allen E. B. (2003a). Nitrogen enrichment alters mycorrhizal allocation at five mesic to semiarid grasslands. Ecology 84, 1895–1908. 10.1890/0012-9658(2003)084[1895:NEAMAA]2.0.CO;2

[B54] Johnson N. C. Wilson G. W. T. Bowker M. A. Wilson J. A. Miller R. M. (2010). Resource limitation is a driver of local adaptation in mycorrhizal symbioses. Proc. Natl. Acad. Sci. U. S. A. 107, 2093–2098. 10.1073/pnas.0906710107 20133855 PMC2836645

[B55] Johnson N. C. Wilson G. W. T. Wilson J. A. Miller R. M. Bowker M. A. (2015). Mycorrhizal phenotypes and the Law of the Minimum. New Phytol. 205, 1473–1484. 10.1111/nph.13172 25417818

[B56] Johnson N. C. Wolf J. Koch G. W. (2003b). Interactions among mycorrhizae, atmospheric CO2 and soil N impact plant community composition. Ecol. Lett. 6, 532–540. 10.1046/j.1461-0248.2003.00460.x

[B57] Johnson N. Copeland P. Crookston R. Pfleger F. (1992). Mycorrhizae: possible explanation for yield decline with continuous corn and soybean. Agron 84, 387–390. 10.2134/agronj1992.00021962008400030007x

[B58] Kiers E. T. Denison R. F. (2008). Sanctions, cooperation and the stability of plant–rhizosphere mutualisms. Annu. Rev. Ecol. Evol. Syst. 39, 215–236. 10.1146/annurev.ecolsys.39.110707.173423

[B59] Kiers E. T. Rousseau R. A. West S. A. Denison R. F. (2003). Host sanctions and the legume–rhizobium mutualism. Nature 425, 78–81. 10.1038/nature01931 12955144

[B60] Kiers T. E. Duhamel M. Beesetty Y. Mensah J. A. Franken O. Verbruggen E. (2011). Reciprocal rewards stabilize cooperation in the mycorrhizal symbiosis. Science 333, 880–882. 10.1126/science.1208473 21836016

[B61] Krohn A. (2016). akutils-v12: facilitating analyses of microbial communities through QIIME. Zenodo. 10, 5281.

[B62] Kulmatiski A. (2018). Community-level plant–soil feedbacks explain landscape distribution of native and non-native plants. Ecol. Evol. 8, 2041–2049. 10.1002/ece3.3649 29468023 PMC5817120

[B63] Larimer A. L. Bever J. D. Clay K. (2010). The interactive effects of plant microbial symbionts: a review and meta-analysis. Symbiosis 51, 139–148. 10.1007/s13199-010-0083-1

[B64] Lee J. Lee S. Young J. P. W. (2008). Improved PCR primers for the detection and identification of arbuscular mycorrhizal fungi. FEMS Microbiol. Ecol. 65, 339–349. 10.1111/j.1574-6941.2008.00531.x 18631176

[B65] Levy-Booth D. J. Prescott C. E. Grayston S. J. (2014). Microbial functional genes involved in nitrogen fixation, nitrification and denitrification in forest ecosystems. Soil Biol. Biochem. 75, 11–25. 10.1016/j.soilbio.2014.03.021

[B66] Love M. Anders S. Huber W. (2014). Differential analysis of count data–the DESeq2 package. Genome Biol. 15 (550), 10–1186. 110.1186/s13059-014-0550-8 10.1186/s13059-014-0550-8 PMC4302049 25516281

[B67] Lozupone C. Knight R. (2005). UniFrac: a new phylogenetic method for comparing microbial communities. Appl. Environ. Microbiol. 71, 8228–8235. 10.1128/AEM.71.12.8228-8235.2005 16332807 PMC1317376

[B68] Lugtenberg B. Kamilova F. (2009). Plant-growth-promoting rhizobacteria. Annu. Rev. Microbiol. 63, 541–556. 10.1146/annurev.micro.62.081307.162918 19575558

[B69] Mahé F. Rognes T. Quince C. de Vargas C. Dunthorn M. (2014). Swarm: robust and fast clustering method for amplicon-based studies. PeerJ 2, e593. 10.7717/peerj.593 25276506 PMC4178461

[B70] Mangan M. E. Sheaffer C. Wyse D. L. Ehlke N. J. Reich P. B. (2011). Native perennial grassland species for bioenergy: establishment and biomass productivity. Agron. J. 103, 509–519. 10.2134/agronj2010.0360

[B71] Mao Y. Li X. Smyth E. M. Yannarell A. C. Mackie R. I. (2014). Enrichment of specific bacterial and eukaryotic microbes in the rhizosphere of switchgrass (*Panicum virgatum* L.) through root exudates. Environ. Microbiol. Rep. 6, 293–306. 10.1111/1758-2229.12152 24983534

[B72] Mariotte P. Canarini A. Dijkstra F. A. (2017). Stoichiometric N: P flexibility and mycorrhizal symbiosis favour plant resistance against drought. J. Ecol. 105(4), 958–967. 10.1111/1365-2745.12731

[B73] Martiny A. C. Treseder K. Pusch G. (2013). Phylogenetic conservatism of functional traits in microorganisms. ISME J. 7 (4), 830–838. 10.1038/ismej.2012.160 23235290 PMC3603392

[B74] Martiny J. B. Jones S. E. Lennon J. T. Martiny A. C. (2015). Microbiomes in light of traits: a phylogenetic perspective. Science 350 (6261), aac9323. 10.1126/science.aac9323 26542581

[B75] McDonald D. Price M. N. Goodrich J. Nawrocki E. P. DeSantis T. Z. Probst A. (2012). An improved Greengenes taxonomy with explicit ranks for ecological and evolutionary analyses of bacteria and archaea. ISME J. 6, 610–618. 10.1038/ismej.2011.139 22134646 PMC3280142

[B76] Mehlich A. (1984). Mehlich 3 soil test extractant: a modification of Mehlich 2 extractant. Commun. Soil Sci. Plant Anal. 15, 1409–1416. 10.1080/00103628409367568

[B77] Moore J. A. M. Jiang J. Patterson C. M. Mayes M. A. (2015). Interactions among roots, mycorrhizas and free-living microbial communities differentially impact soil carbon processes. J. Ecol. 103, 1442–1453. 10.1111/1365-2745.12484

[B78] Morris G. P. Hu Z. Grabowski P. P. Borevitz J. O. de Graaff M. A. Miller R. M. (2016). Genotypic diversity effects on biomass production in native perennial bioenergy cropping systems. GCB Bioenergy 8, 1000–1014. 10.1111/gcbb.12309 27668013 PMC5019262

[B79] Nemergut D. R. Townsend A. R. Sattin S. R. Freeman K. R. Fierer N. Neff J. C. (2008). The effects of chronic nitrogen fertilization on alpine tundra soil microbial communities: implications for carbon and nitrogen cycling. Environ. Microbiol. 10, 3093–3105. 10.1111/j.1462-2920.2008.01735.x 18764871

[B80] Oates L. G. Duncan D. S. Sanford G. R. Liang C. Jackson R. D. (2016). Bioenergy cropping systems that incorporate native grasses stimulate growth of plant-associated soil microbes in the absence of nitrogen fertilization. Agric. Ecosyst. Environ. 233, 396–403. 10.1016/j.agee.2016.09.008

[B81] Olsson P. A. Bååth E. Jakobsen I. Söderström B. (1995). The use of phospholipid and neutral lipid fatty acids to estimate biomass of arbuscular mycorrhizal fungi in soil. Mycol. Res. 99, 623–629. 10.1016/S0953-7562(09)80723-5

[B82] Öpik M. Vanatoa E. Moora M. Davison J. Kalwij J. M. Reier U. (2010). The online database Maarj AM reveals global and ecosystemic distribution patterns in arbuscular mycorrhizal fungi (Glomeromycota). New Phytol. 188, 223–241. 10.1111/j.1469-8137.2010.03334.x 20561207

[B83] Parrish D. J. Fike J. H. (2005). The biology and agronomy of switchgrass for biofuels. CRC. Crit. Rev. Plant Sci. 24, 423–459. 10.1080/07352680500316433

[B84] Paulson J. N. Stine O. C. Bravo H. C. Pop M. (2013). Differential abundance analysis for microbial marker-gene surveys. Nat. Methods 10, 1200–1202. 10.1038/nmeth.2658 24076764 PMC4010126

[B85] Perlack R. D. Stokes B. J. Eaton L. M. Turnhollow A. F. (2011). US billion-ton update. Biomass supply for a bioenergy and bioproducts industry. Renew. Energy 7, 1–229. 10.1089/ind.2011.7.375

[B86] Philippot L. Andersson S. G. Battin T. J. Prosser J. I. Schimel J. P. Whitman W. B. (2010). The ecological coherence of high bacterial taxonomic ranks. Nat. Rev. Microbiol. 8 (7), 523–529. 10.1038/nrmicro2367 20531276

[B87] Price M. N. Dehal P. S. Arkin A. P. (2009). FastTree: computing large minimum evolution trees with profiles instead of a distance matrix. Mol. Biol. Evol. 26, 1641–1650. 10.1093/molbev/msp077 19377059 PMC2693737

[B88] Prober S. M. Leff J. W. Bates S. T. Borer E. T. Firn J. Harpole W. S. (2015). Plant diversity predicts beta but not alpha diversity of soil microbes across grasslands worldwide. Ecol. Lett. 18, 85–95. 10.1111/ele.12381 25430889

[B89] Püschel D. Janoušková M. Hujslová M. Slavíková R. Gryndlerová H. Jansa J. (2016). Plant–fungus competition for nitrogen erases mycorrhizal growth benefits of *Andropogon gerardii* under limited nitrogen supply. Ecol. Evol. 6, 4332–4346. 10.1002/ece3.2207 27386079 PMC4930984

[B90] Ramirez K. S. Craine J. M. Fierer N. (2012). Consistent effects of nitrogen amendments on soil microbial communities and processes across biomes. Glob. Chang. Biol. 18, 1918–1927. 10.1111/j.1365-2486.2012.02639.x

[B91] Reed S. C. Cleveland C. C. Townsend A. R. (2011). Functional ecology of free-living nitrogen fixation: a contemporary perspective. Annu. Rev. Ecol. Evol. Syst. 42, 489–512. 10.1146/annurev-ecolsys-102710-145034

[B92] Revillini D. (2018). The role of shifting resource availability in shaping grassland-soil microbial symbiont interactions.

[B93] Revillini D. Gehring C. A. Johnson N. C. (2016). The role of locally adapted mycorrhizas and rhizobacteria in plant–soil feedback systems. Funct. Ecol. 30, 1086–1098. 10.1111/1365-2435.12668

[B94] Reynolds H. L. Hartley A. E. Vogelsang K. M. Bever J. D. Schultz P. A. (2005). Arbuscular mycorrhizal fungi do not enhance nitrogen acquisition and growth of old-field perennials under low nitrogen supply in glasshouse culture. New Phytol. 167, 869–880. 10.1111/j.1469-8137.2005.01455.x 16101923

[B95] Rognes T. Flouri T. Nichols B. Quince C. Mahé F. (2016). VSEARCH: a versatile open source tool for metagenomics. PeerJ 4, e2584. 10.7287/peerj.preprints.2409v1 27781170 PMC5075697

[B96] Rubin R. L. Groenigen K. J. Hungate B. A. (2017). Plant growth promoting rhizobacteria are more effective under drought: a meta-analysis. Plant Soil. 416 (1–2), 309–323. 10.1007/s11104-017-3199-8

[B97] Sakamoto K. Iijima T. Higuchi R. (2004). Use of specific phospholipid fatty acids for identifying and quantifying the external hyphae of the arbuscular mycorrhizal fungus *Gigaspora rosea* . Soil Biol. Biochem. 36, 1827–1834. 10.1016/j.soilbio.2004.04.037

[B98] Schlaeppi K. Bulgarelli D. (2015). The plant microbiome at work. Mol. Plant–Microbe Interact. 28, 212–217. 10.1094/MPMI-10-14-0334-FI 25514681

[B99] Semchenko M. Leff J. W. Lozano Y. M. Saar S. Davison J. Wilkinson A. (2018). Fungal diversity regulates plant–soil feedbacks in temperate grassland. Sci. Adv. 4, eaau4578. 10.1126/sciadv.aau4578 30498781 PMC6261650

[B100] Sharma M. P. Buyer J. S. (2015). Comparison of biochemical and microscopic methods for quantification of arbuscular mycorrhizal fungi in soil and roots. Appl. Soil Ecol. 95, 86–89. 10.1016/j.apsoil.2015.06.001

[B101] Shen C. Ge Y. Yang T. Chu H. (2017). Verrucomicrobial elevational distribution was strongly influenced by soil pH and carbon/nitrogen ratio. J. Soils Sediments 17, 2449–2456. 10.1007/s11368-017-1680-x

[B102] Singer E. Bonnette J. Kenaley S. C. Woyke T. Juenger T. E. (2019). Plant compartment and genetic variation drive microbiome composition in switchgrass roots. Environ. Microbiol. Rep. 11, 185–195. 10.1111/1758-2229.12727 30537406 PMC6850097

[B103] Sprunger C. D. Oates L. G. Jackson R. D. Robertson G. P. (2017). Plant community composition influences fine root production and biomass allocation in perennial bioenergy cropping systems of the upper Midwest, USA. Biomass Bioenergy 105, 248–258. 10.1016/j.biombioe.2017.07.007

[B104] Strickland M. S. Rousk J. (2010). Considering fungal:bacterial dominance in soils—methods, controls, and ecosystem implications. Soil Biol. Biochem. 42, 1385–1395. 10.1016/j.soilbio.2010.05.007

[B105] Tilman D. Hill J. Lehman C. (2006). Carbon-negative biofuels from low-input high-diversity grassland biomass. Science 314, 1598–1600. 10.1126/science.1133306 17158327

[B106] Toju H. Peay K. G. Yamamichi M. Narisawa K. Hiruma K. Naito K. (2018). Core microbiomes for sustainable agroecosystems. Nat. Plants 4, 247–257. 10.1038/s41477-018-0139-4 29725101

[B107] Treusch A. H. Leininger S. Kietzin A. Schuster S. C. Klenk H. P. Schleper C. (2005). Novel genes for nitrite reductase and Amo-related proteins indicate a role of uncultivated mesophilic crenarchaeota in nitrogen cycling. Environ. Microbiol. 7, 1985–1995. 10.1111/j.1462-2920.2005.00906.x 16309395

[B108] Vacher C. Hampe A. Porté A. J. Sauer U. Compant S. Morris C. E. (2016). The phyllosphere: microbial jungle at the plant–climate interface. Annu. Rev. Ecol. Evol. Syst. 47, 1–24. 10.1146/annurev-ecolsys-121415-032238

[B109] van der Heijden M. G. Bruin S. Luckerhoff L. van Logtestijn R. S. Schlaeppi K. (2016). A widespread plant–fungal–bacterial symbiosis promotes plant biodiversity, plant nutrition and seedling recruitment. ISME J. 10, 389–399. 10.1038/ismej.2015.120 26172208 PMC4737930

[B110] van der Putten W. H. Bardgett R. D. Bever J. D. Bezemer T. M. Casper B. B. Fukami T. (2013). Plant–soil feedbacks: the past, the present and future challenges. J. Ecol. 101, 265–276. 10.1111/1365-2745.12054

[B111] Vandenkoornhuyse P. Quaiser A. Duhamel M. Le Van A. Dufresne A. (2015). The importance of the microbiome of the plant holobiont. New Phytol. 206, 1196–1206. 10.1111/nph.13312 25655016

[B112] Verbruggen E. Xiang D. Chen B. Xu T. Rillig M. C. (2015) Mycorrhizal fungi associated with high soil N:P ratios are more likely to be lost upon conversion from grasslands to arable agriculture. Soil Biol. Biochem. 86, 1–4. 10.1016/j.soilbio.2015.03.008

[B113] Wardle D. A. (2006). The influence of biotic interactions on soil biodiversity. Ecol. Lett. 9, 870–886. 10.1111/j.1461-0248.2006.00931.x 16796577

[B114] Wei C. Yu Q. Bai E. Lü X. Li Q. Xia J. (2013). Nitrogen deposition weakens plant–microbe interactions in grassland ecosystems. Glob. Chang. Biol. 19, 3688–3697. 10.1111/gcb.12348 23925948

[B115] Wilsey B. J. Wayne Polley H. (2006). Aboveground productivity and root–shoot allocation differ between native and introduced grass species. Oecologia 150, 300–309. 10.1007/s00442-006-0515-z 16927104

[B116] Wright L. Turhollow A. (2010). Switchgrass selection as a “model” bioenergy crop: a history of the process. Biomass Bioenergy 34, 851–868. 10.1016/j.biombioe.2010.01.030

[B117] Yang Y. Tilman D. Lehman C. Trost J. J. (2018). Sustainable intensification of high-diversity biomass production for optimal biofuel benefits. Nat. Sustain. 11, 686. 10.1038/s41893-018-0166-1

[B118] Zehr J. P. Jenkins B. D. Short S. M. Steward G. F. (2003). Nitrogenase gene diversity and microbial community structure: a cross-system comparison. Environ. Microbiol. 5, 539–554. 10.1046/j.1462-2920.2003.00451.x 12823187

[B119] Zheng C. Ji B. Zhang J. Zhang F. Bever J. D. (2014). Shading decreases plant carbon preferential allocation towards the most beneficial mycorrhizal mutualist. New Phytol. 205, 361–368. 10.1111/nph.13025 25243653

[B120] Zhou X. Fornara D. Anne E. Wang D. Ren G. Christie P. (2015). Effects of 44 years of chronic nitrogen fertilization on the soil nitrifying community of permanent grassland. Soil Biol. Biochem. 91, 76–83. 10.1016/j.soilbio.2015.08.031

[B121] Zogg G. P. Zak D. R. Ringelberg D. B. White D. C. MacDonald N. W. Pregitzer K. S. (1997). Compositional and functional shifts in microbial communities due to soil warming. Soil Sci. Soc. Am. J. 61, 475. 10.2136/sssaj1997.03615995006100020015x

