# Supplementary material for: Plant Diversity and Fertilizer Management Shape the Belowground Microbiome of Native Grass Bioenergy Feedstocks
Source: Front Plant Sci. 2019 Aug 14;10:1018. doi: 10.3389/fpls.2019.01018 (PMC6702339; doi:10.3389/fpls.2019.01018)
Supplement: Supplementary file 1 [file DataSheet_1.pdf]

Supplemental Table 1. Plant taxa, cultivar, functional group, and seeding rates in the prairie mixture treatment.

Species	Cultivar	Group	Prairie mixture (seeding%)
Panicum virgatum		C4	20
	Cave-in-rock		7
	Kanlow		7
	Southlow		7
Andropogon gerardii		C4	20
	Roundtree		7
	Epic		7
	Southlow		7
Sorghastrum nutans		C4	20
Elymus canadensis		C3	20
Desmodium canadense		Legume	2.5
Lespedeza capitata		Legume	2.5
Dalea purpurea		Legume	2.5
Aster nava-angliae		Composite	2.5
Coreopsis tripteris		Composite	2.5
Heliopsis helianthoides		Composite	2.5
Ratibida pinnata		Composite	2.5
Veronicastrum virginicum		Forb	2.5
