# Supplementary material for: Plant Diversity and Fertilizer Management Shape the Belowground Microbiome of Native Grass Bioenergy Feedstocks
Source: Front Plant Sci. 2019 Aug 14;10:1018. doi: 10.3389/fpls.2019.01018 (PMC6702339; doi:10.3389/fpls.2019.01018)
Supplement: Supplementary file 2 [file DataSheet_2.pdf]

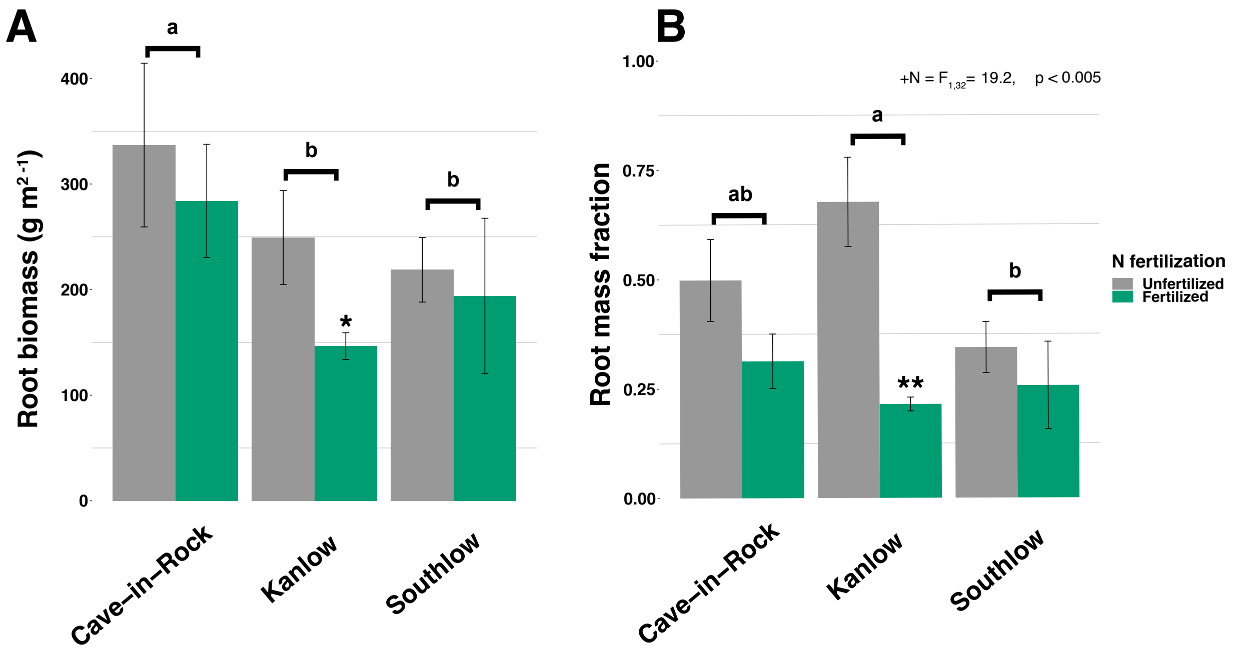

Supplementary Figure 1. Root biomass (A) and root mass fraction (B) from each planting mixture under unfertilized or N-fertilized conditions. Letters indicate significant differences from Tukey's HSD post-hoc testing between treatments, asterisks indicate significant differences from ANOVA within treatments (* = $p < 0.05$, ** = $p < 0.01$).
