# Supplementary material for: Plant Diversity and Fertilizer Management Shape the Belowground Microbiome of Native Grass Bioenergy Feedstocks
Source: Front Plant Sci. 2019 Aug 14;10:1018. doi: 10.3389/fpls.2019.01018 (PMC6702339; doi:10.3389/fpls.2019.01018)
Supplement: Supplementary file 3 [file DataSheet_3.pdf]

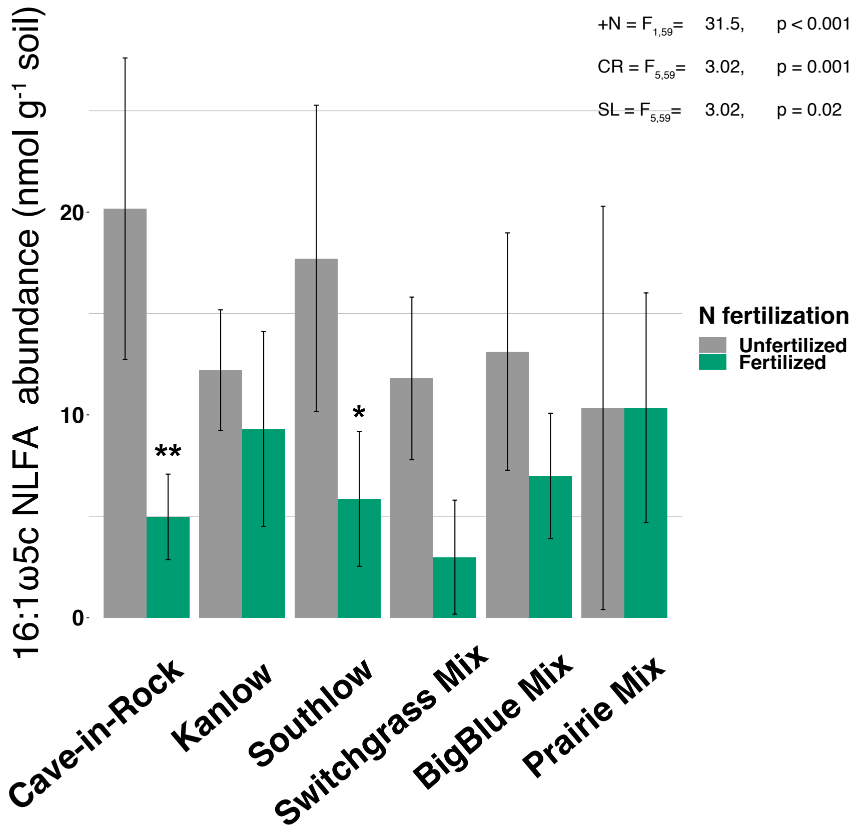

Supplementary Figure 2. Total AM fungal biomass (16:15c NLFA) from soils of each planting mixture under unfertilized or N-fertilized conditions. N fertilization significantly decreased AM fungal biomass for Cave-in-Rock and Southlow. Asterisks indicate significant different from ANOVA within treatments (* = $p < 0.05$, ** = $p < 0.01$).
