# Supplementary material for: Plant Diversity and Fertilizer Management Shape the Belowground Microbiome of Native Grass Bioenergy Feedstocks
Source: Front Plant Sci. 2019 Aug 14;10:1018. doi: 10.3389/fpls.2019.01018 (PMC6702339; doi:10.3389/fpls.2019.01018)
Supplement: Supplementary file 4 [file DataSheet_4.pdf]

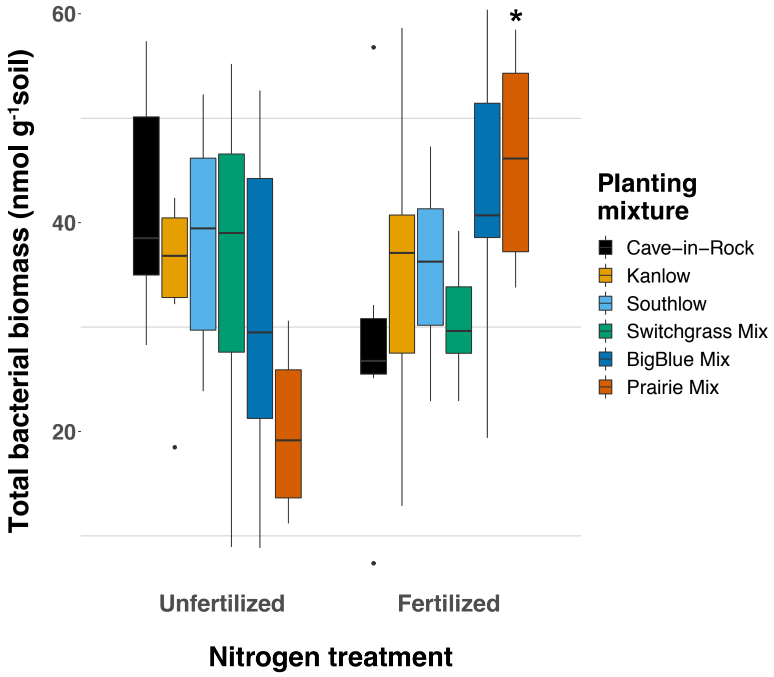

Supplementary Figure 3. Total bacterial PLFA biomass from unfertilized and +N treatments, colored by planting mixture. Asterisk indicates significant differences from ANOVA after fertilization, within a planting mixture ($p < 0.05$).
