# Supplementary material for: Plant Diversity and Fertilizer Management Shape the Belowground Microbiome of Native Grass Bioenergy Feedstocks
Source: Front Plant Sci. 2019 Aug 14;10:1018. doi: 10.3389/fpls.2019.01018 (PMC6702339; doi:10.3389/fpls.2019.01018)
Supplement: Supplementary file 5 [file DataSheet_5.pdf]

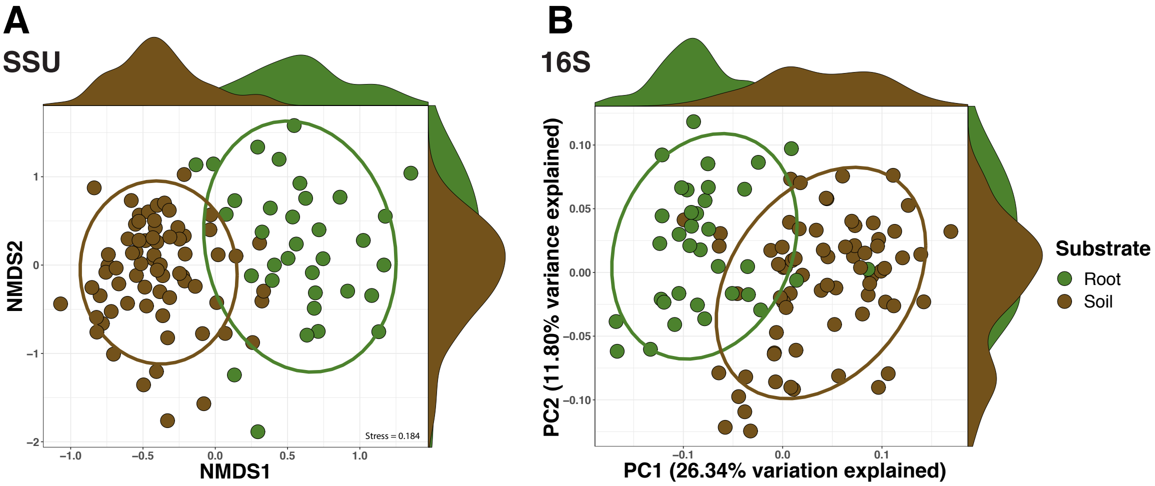

Supplementary Figure 4. NMDS of AM fungal community Bray-Curtis dissimilarity (A) and bacterial weighted UniFrac (B) in roots (green) and soil (brown). Value densities for each substrate are on respective axes.
