# Supplementary material for: Plant Diversity and Fertilizer Management Shape the Belowground Microbiome of Native Grass Bioenergy Feedstocks
Source: Front Plant Sci. 2019 Aug 14;10:1018. doi: 10.3389/fpls.2019.01018 (PMC6702339; doi:10.3389/fpls.2019.01018)
Supplement: Supplementary file 6 [file DataSheet_6.pdf]

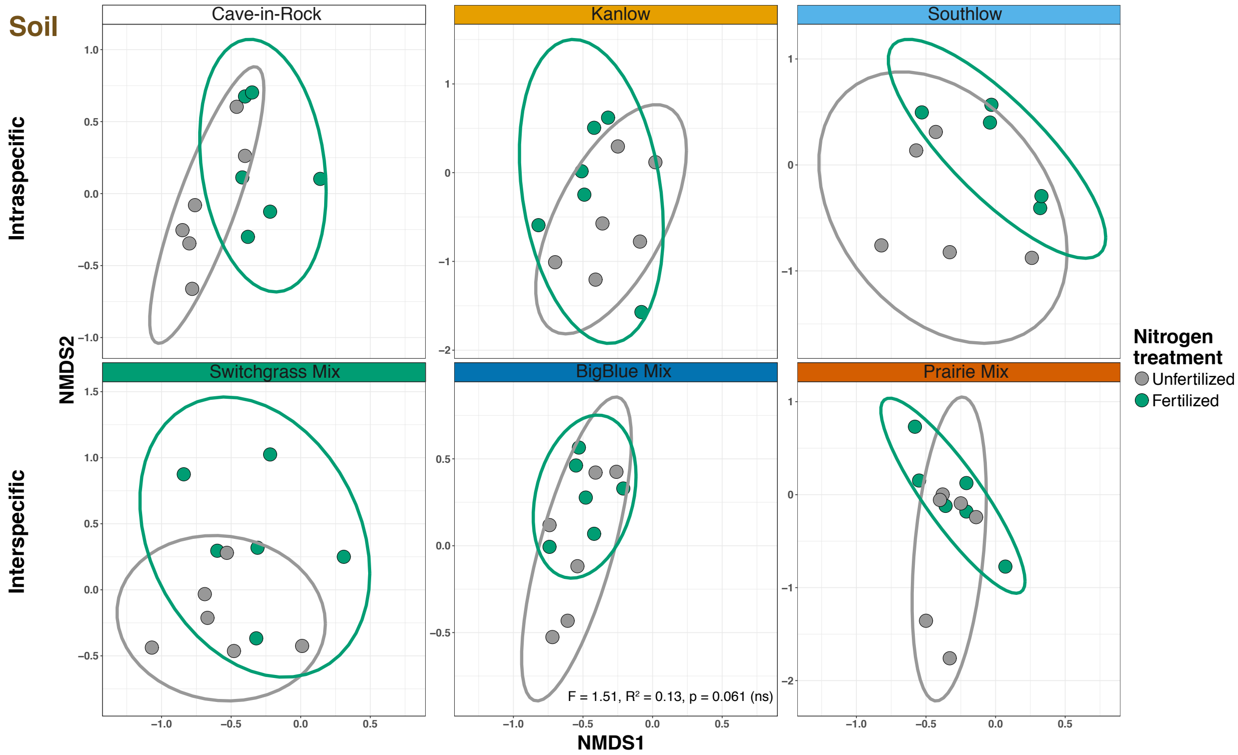

Supplementary Figure 5. NMDS of soil AM fungal community Bray-Curtis dissimilarity, with panels for each planting mixture and colored by N fertilization treatment. Ellipses represent 95% confidence areas around respective N fertilization treatment.
