# Supplementary material for: Plant Diversity and Fertilizer Management Shape the Belowground Microbiome of Native Grass Bioenergy Feedstocks
Source: Front Plant Sci. 2019 Aug 14;10:1018. doi: 10.3389/fpls.2019.01018 (PMC6702339; doi:10.3389/fpls.2019.01018)
Supplement: Supplementary file 8 [file DataSheet_8.pdf]

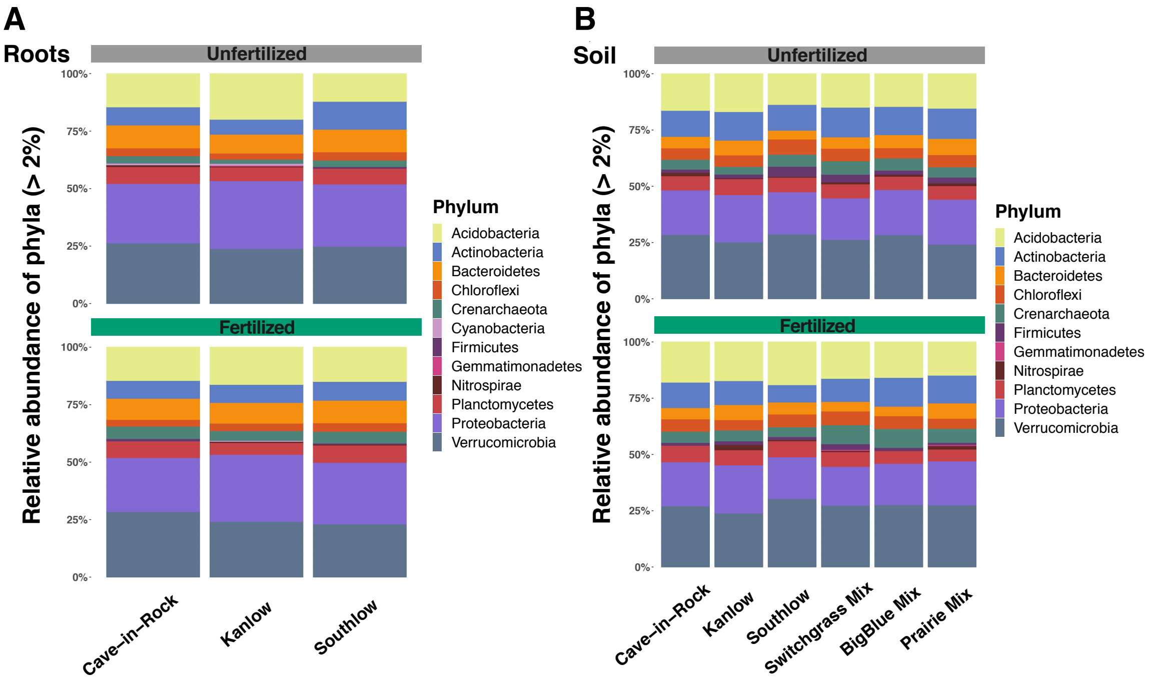

Supplementary Figure 7. Relative abundance of bacterial phyla on roots (A) or in soils (B) under unfertilized or N-fertilized plots for all planting mixtures. Rare taxa have been removed, leaving only those that contributed more than 2% relative abundance to each sample.
