# Supplementary material for: Plant Diversity and Fertilizer Management Shape the Belowground Microbiome of Native Grass Bioenergy Feedstocks
Source: Front Plant Sci. 2019 Aug 14;10:1018. doi: 10.3389/fpls.2019.01018 (PMC6702339; doi:10.3389/fpls.2019.01018)
Supplement: Supplementary file 9 [file DataSheet_9.pdf]

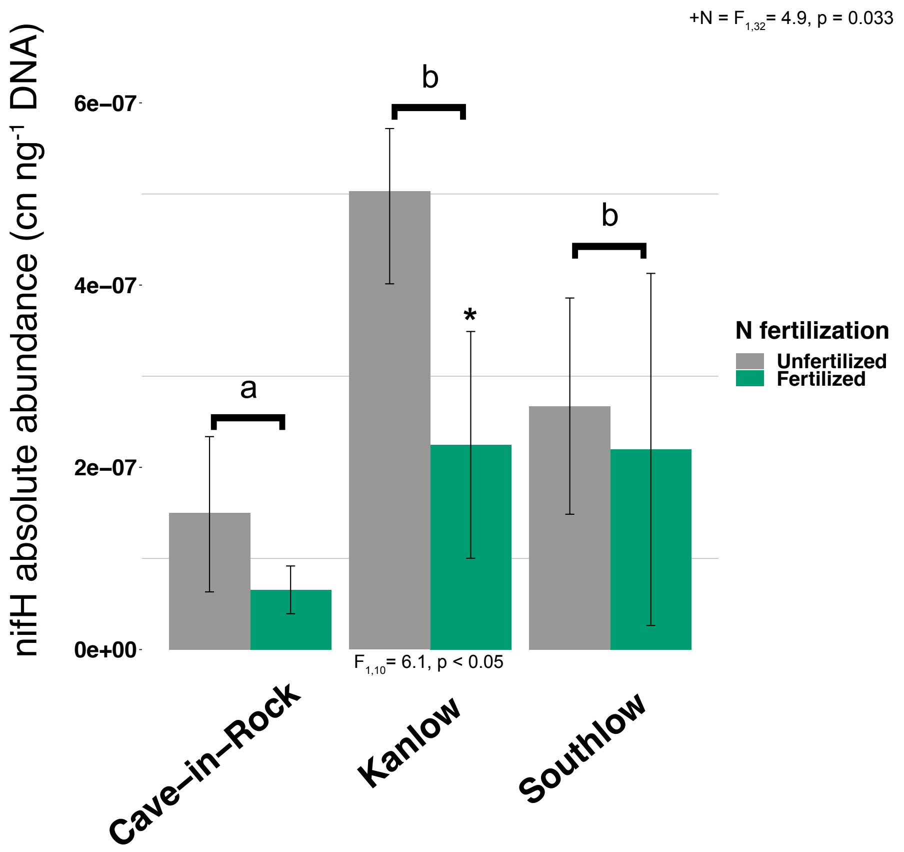

Supplementary Figure 8. Total nifH gene abundance from each planting mixture under unfertilized or N-fertilized conditions. Letters indicate significant differences from Tukey's HSD post-hoc testing, asterisks indicate significant different from ANOVA (* = $p < 0.05$, ** = $p < 0.01$).
