# Supplementary material for: Plant Diversity and Fertilizer Management Shape the Belowground Microbiome of Native Grass Bioenergy Feedstocks
Source: Front Plant Sci. 2019 Aug 14;10:1018. doi: 10.3389/fpls.2019.01018 (PMC6702339; doi:10.3389/fpls.2019.01018)
Supplement: Supplementary file 10 [file DataSheet_10.pdf]

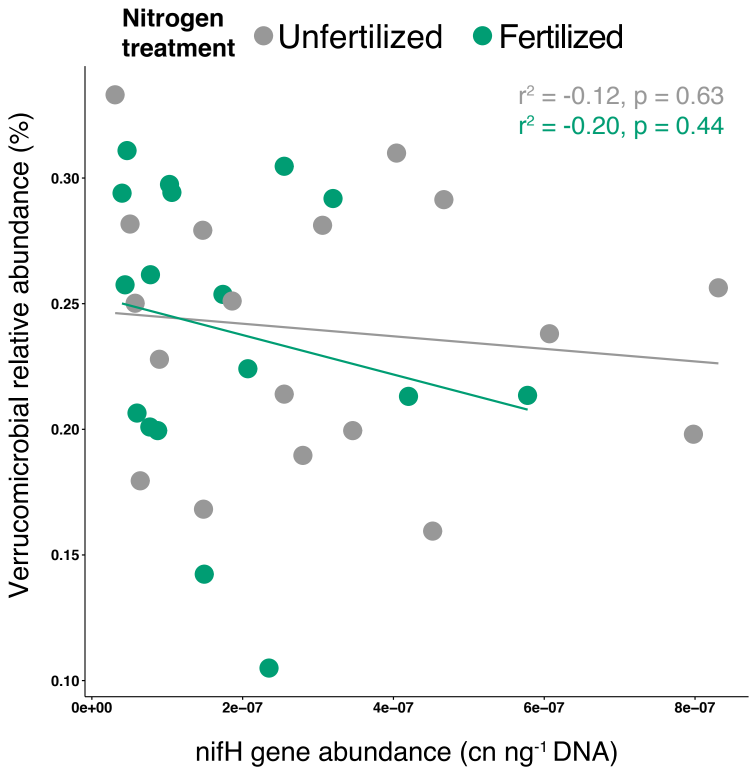

Supplementary Figure 9. Relationship between relative abundance of *Verrucomicrobia* on roots and total nifH gene abundance on roots, colored by nitrogen fertilization treatment. There were no significant correlations. Statistics are provided in the upper right corner for both unfertilized (gray) and N-fertilized (green) treatments.
